# Usability and Preliminary Efficacy of an Artificial Intelligence–Driven Platform Supporting Dietary Management in Diabetes: Mixed Methods Study

**DOI:** 10.2196/43959

**Published:** 2023-08-09

**Authors:** Kim Bul, Nikki Holliday, Mohammad Rashed Alam Bhuiyan, Cain C T Clark, John Allen, Petra A Wark

**Affiliations:** 1 Research Institute for Health and Wellbeing Coventry University Coventry United Kingdom; 2 Centre for Trust Peace and Social Relations Coventry University Coventry United Kingdom; 3 Department of Political Sciences University of Dhaka Dhaka Bangladesh; 4 Warwickshire InStitute for Diabetes, Endocrinology & Metabolism University Hospitals Coventry & Warwickshire NHS Trust Coventry United Kingdom

**Keywords:** nutrition and dietetics, general diabetes, qualitative research, preventive medicine, web technology, self-management, diabetes, nutrition, deep learning, artificial, mobile phone

## Abstract

**Background:**

Nutrition plays an important role in diabetes self-management. Web-based diabetes care, driven by artificial intelligence (AI), enables more personalized care.

**Objective:**

This study aimed to examine the usability and preliminary efficacy of a web-based AI-driven nutrition platform to support people with diabetes and their carers in identifying healthy recipes, meal planning, and web-based shopping.

**Methods:**

Diabetes UK signposted people with diabetes and their carers to the platform’s study-specific portal through its website, social media, and newsletters. A total of 73 adult participants with prediabetes or diabetes or their carers completed the baseline web-based survey. Of these 73 participants, 23 (32%) completed a web-based survey after 8 weeks of platform use. Web-based semistructured interviews were conducted with platform users (7/23, 30%) who agreed to be followed up and diabetes experts (n=3) who had nutrition and platform knowledge. The intervention consists of a web-based platform that incorporates AI to personalize recipes, meal planning, and shopping list experiences and was made available for 8 weeks. Baseline characteristics, satisfaction, system usability, and diabetes-related and general health indicators were assessed before and after using the platform for 8 weeks.

**Results:**

Reductions in weight (mean difference 4.5 kg/m^2^, 95% CI 1.0-12.0; *P*=.009; Cliff δ=0.33) and waist size (mean difference 3.9 cm, 95% CI 2.0-6.5; *P*=.008; Cliff δ=0.48) were found. Most of the participants (151/217, 69.6%) did not regularly use the platform and had low or very low engagement scores. However, the platform was perceived as accessible with no need for additional assistance (11/21, 52%), user-friendly (8/21, 38%), and easy to use (8/21, 38%), regardless of some usability issues. Saving recipes was the most popular feature, with 663 saved recipes.

**Conclusions:**

This study indicated that the usability of the nutrition platform was well perceived by users and their carers. As participants managed their diabetes well, adding an education component would be specifically relevant for people less familiar with the role of diet in diabetes management. To assess the platform’s effectiveness in improving diabetes-related health indicators, controlled studies with a larger and more diverse participant sample are recommended.

## Introduction

### Background

The prevalence of diabetes mellitus, one of the most burdensome noncommunicable diseases, has been rising as have its mortality rates and adverse societal and economic consequences [1-3]. Worldwide prevalence rates are as high as 9.3% (463 million people), with an expected increase to 10.9% (700 million people) by 2045 [4]. In 2018-2019, a total of 3.9 million people were diagnosed with diabetes in the United Kingdom. Diabetes can have severe health complications, including loss of eyesight, kidney disease, hypertension, heart failure, and diabetic feet, with rising economic costs for the National Health Service as high as £9.8 billion (US $12.4 billion) per year [5].

Type 2 diabetes is the most common form of diabetes, and it is associated with an unhealthy lifestyle in terms of physical exercise, nutrition, and weight. To manage diabetes in the long term, a combination of a healthy lifestyle and medication seems optimal. However, as individual factors (eg, comorbidities) can make glycemic control (glycated hemoglobin [HbA_1c_] levels) through medication challenging, a change in lifestyle is preferred as a first-step approach [6]. Under ideal circumstances, type 2 diabetes can be reversed, as demonstrated in a clinical trial where up to 46% and 36% of the patients had successfully reversed type 2 diabetes at 12 and 24 months after diagnosis, respectively [7]. In people with type 1 diabetes, there is a greater emphasis on counting carbohydrates and calories, but lifestyle management seems just as important to manage HbA_1c_ levels and prevent health complications (eg, cardiometabolic risk) [8].

On the basis of the results of a systematic review and meta-analysis [9], lifestyle interventions seemed to reduce the incidence of type 2 diabetes and improve glycemic outcomes, anthropometric measures, physical activity, and energy intake across an ethnically diverse sample of adults at risk for developing type 2 diabetes compared with a control group. Focusing on lifestyle factors such as nutrition and physical activity does seem a promising avenue for prevention of type 2 diabetes in individuals considered to be at high risk.

Websites, mobile apps, artificial intelligence (AI) systems, serious games, automated calls and messages, and medical devices for diabetes prevention and care have gained popularity [10,11], especially during and after the COVID-19 pandemic, with the aim to improve care accessibility and self-management [12]. An overview of 15 systematic reviews showed that mobile health (mHealth) interventions can be effective in improving HbA_1c_ levels, specifically for people with type 2 diabetes; however, the methodological quality of most of the reviews was limited [13]. A systematic review published later indicated that mHealth interventions have the potential to reduce weight, but the study findings, outcomes, and intervention durations were very heterogeneous [12]. A web-based education program offering support on nutritional management that was available for people with type 2 diabetes (or those with prediabetes) and their carers resulted in improved nutritional knowledge and people’s intentions to eat healthier and follow a healthy lifestyle [14].

### Objectives

Most mHealth and health apps focus on monitoring blood glucose levels and have some educational components [12] (eg, by providing an AI-based embodied conversational agent that educates patients with type 2 diabetes about self-management) [15]. However, none of these digital apps used recipe exchange, meal planning, and web-based shopping features as catalysts for a healthy lifestyle and thereby diabetes management. This study aimed to examine the usability and preliminary efficacy of a web-based nutrition platform (using AI) that is freely accessible to anyone looking for support in identifying healthy recipes, meal planning, and web-based food shopping. The display of nutritional values is relevant for diabetes management, and this has been incorporated across all recipes on the AI-driven web-based platform. Although the application has not been specifically developed for people with diabetes and their carers, we hypothesized that the use of this nutrition platform will improve people’s general and diabetes-related health indicators, diet, and confidence regarding diabetes management.

## Methods

### Design

This mixed methods study had a pretest-posttest design. Quantitative data were derived from a web-based semistructured survey administered to people with diabetes (or their carers) and diabetes experts. Descriptive and inferential statistics (where applicable) were reported for participants’ general and diabetes-related health indicators before and after using the platform for 8 weeks. Platform data were captured with Mixpanel software [16] to assess real-time platform use.

### Participants

The website of the charity Diabetes UK referred potential participants to the platform’s study-specific portal. People with type 1 or type 2 diabetes and those with prediabetes as well as those caring for someone with diabetes were eligible. Participants also had to be aged ≥18 years and have a good understanding of written and spoken English. A selection of the participants who consented to be approached for follow-up research were invited for semistructured interviews after 8 weeks of platform use. Diabetes experts were invited based on their nutrition and platform knowledge.

### AI-Driven Web-Based Nutrition Platform

**Figure 1:**
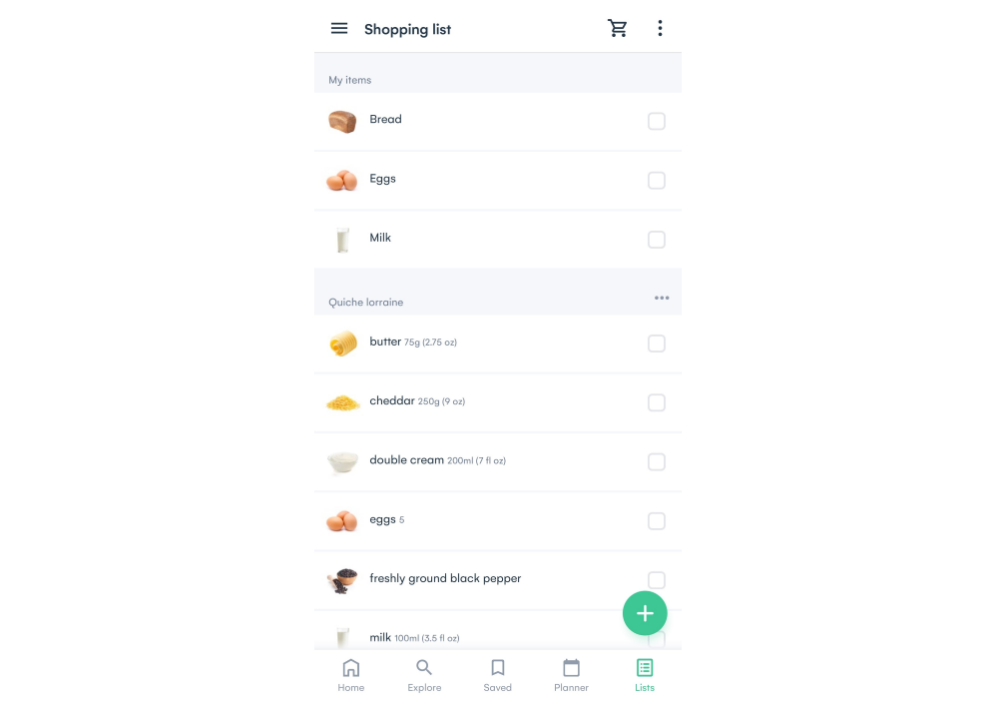
Screenshot of the platform showing the shopping list feature.

**Figure 2:**
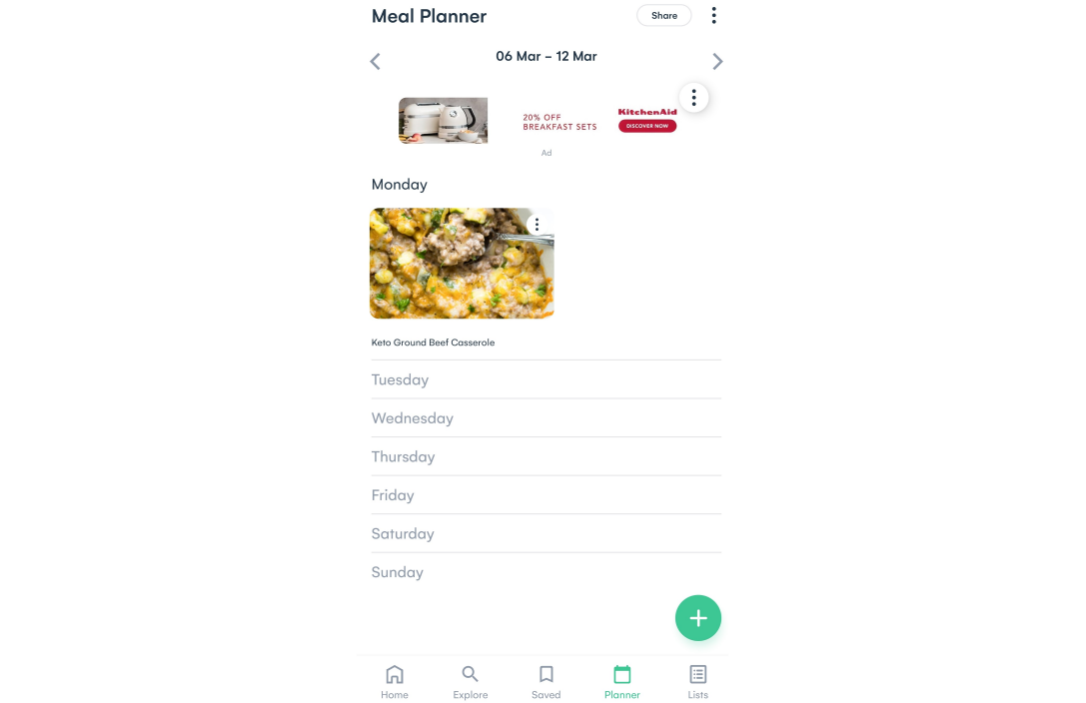
Screenshot of the platform showing the meal planner feature.

**Figure 3:**
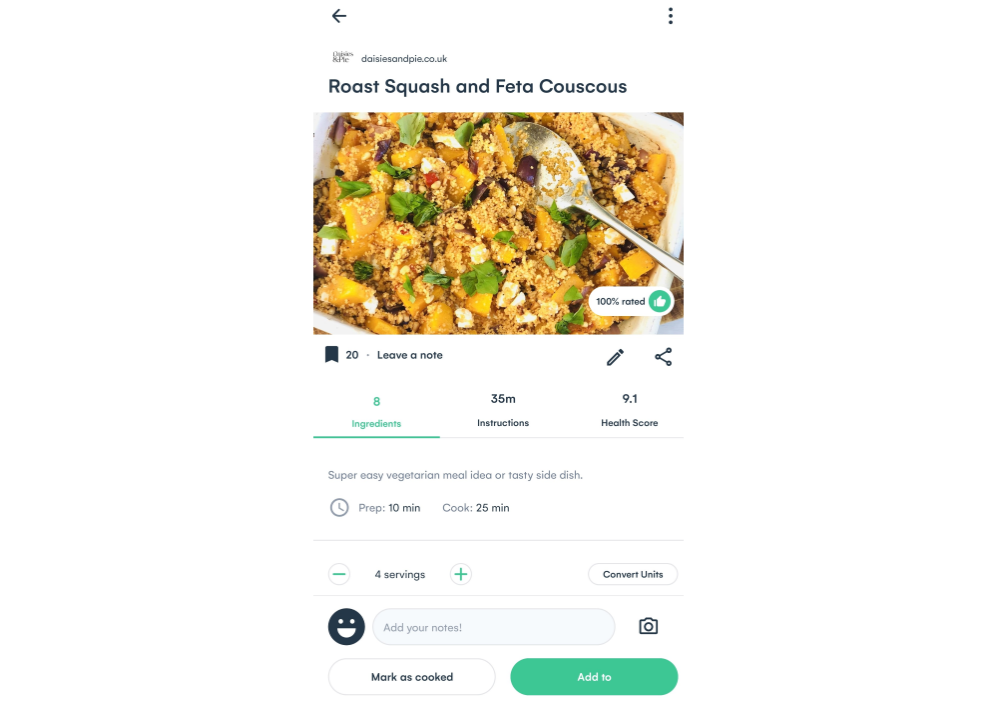
Screenshot of the platform showing the recipe feature.

The platform uses AI to create an ecosystem for users that provides a better journey from recipe inspiration, meal planning, and food item shopping to (web-based) supermarket purchases. The AI software is informed by behavior (eg, viewed, shared, shopped, and saved recipes), preferences (eg, health metrics, family size, diet, avoidances, favorite dishes, and like and dislike ingredients), and context (eg, weather, supermarket deals, user inventory, popular and trending recipes on the web, and food events). It generates personalized recipe suggestions, meal plans, shopping list items, and purchase options based on this information. Powered by deep learning and natural language processing using a natural language–based algorithm, the platform connects millions of data points about ingredients and their relationships to other ingredients, as well as recipe properties (eg, nutritional value, perishability, flavor, and category), including budget and availability across different supermarkets, to ensure good user experience within this ecosystem. Figures 1-3 present different features of the AI-driven web-based nutrition platform.

### Patient and Public Involvement Statement

This study did not involve patients or members of the public, but Diabetes UK represents people with diabetes and their carers, and thereby patient and public members as research participants.

### Procedures

Participants were recruited from September 2020 to April 2021 through the charity Diabetes UK, which signposted participants to the platform’s study-specific portal. They could then sign up for access to the platform. Participants who provided informed consent for study enrollment were invited to take part in a web-based semistructured survey distributed through Qualtrics XM software [17] before and after using the platform for 8 weeks. A maximum of 2 electronic reminders (with a 1-week interval) were sent out where needed. Participants could indicate if they were happy be contacted for a web-based follow-up semistructured (up to 1 hour) or case study (up to 1.5 hours) interview, with the latter contributing to the creation of in-house personas. Diabetes experts from Diabetes UK were invited to participate in a semistructured interview. Data from both interview types have been merged and not presented separately owing to the similarity of identified key themes. Participants received a gift voucher of £10 (US $13.11; semistructured interview) or £20 (US $26.22; semistructured case study interview) to thank them for their time, whereas survey participants could participate in a prize draw (worth £55 [US $72.05]). The Strengthening the Reporting of Observational Studies in Epidemiology (STROBE) checklist for cross-sectional studies has been used for this study reporting [18] (refer to Multimedia Appendix 1 [18] for the completed STROBE checklist).

### Ethics Approval

The study was approved by the Faculty of Health and Life Sciences Ethics Committee at Coventry University (P109725) before study commencement (refer to Multimedia Appendix 2 for the original protocol for the study).

### Assessments

#### Participant Characteristics and Device Use

Sociodemographic characteristics, disability, and smoking status were assessed through a web-based survey sent to participants after enrollment. The survey also asked about the kind of device and internet connection participants anticipated using to access the platform; for which purpose they currently use their device the most; and whether they were already using applications to monitor or improve physical activity, diet, or blood glucose levels. All questions provided the option “Prefer not to say.”

#### Computer Proficiency

The Computer Proficiency Questionnaire-12 (CPQ-12) was used at study commencement. Six domains—computer basics, printing, communication, internet, scheduling, and multimedia—were scored on a 5-point Likert scale ranging from 1=*never tried* to 5=*very easily*. The psychometric properties of the CPQ-12 have been shown to be comparable with those of the longer, 33-question version and were interpreted as excellent [19].

#### General Health Status

The EuroQol Visual Analog Scale (EQ-VAS) was administered before and after participants had used the platform for 8 weeks to measure their current general health status on a scale from 0 (the worst health imagined) to 100 (the best health imagined), with a score of 50 representing the population *average* [20]. The psychometric characteristics of the EQ-VAS have been described as satisfactory in people with diabetes [21].

#### Diabetes-Related Health Indicators

Height (measured in meters and centimeters or feet and inches), weight (kilograms or stones and pounds), waist circumference (centimeters or inches), blood glucose level (HbA_1c_: millimoles per mole), systolic and diastolic blood pressure (millimeters of mercury), and high-density lipoprotein (HDL) and total cholesterol levels (millimoles per liter) were self-reported and assessed before and after participants had access to the platform for 8 weeks. In addition, participants were asked when and by whom the last measurement took place. All questions provided the options “Prefer not to say” and “I don’t know.” Weight (kilograms) was divided by the square of the height (meters) to calculate BMI, which was categorized into underweight (<18.5 kg/m²), healthy weight (18.5-24.9 kg/m²), overweight (25.0-29.9 kg/m²), and obese (≥30.0 kg/m²).

#### Healthy Eating

Participants answered 8 questions about their diet before and after using the platform for 8 weeks, which were derived from the subscales *Food frequency consumption* and *Food habits* of an existing dietary questionnaire [22]. The questions provided an indication of eating habits relevant to people with diabetes and covered variation in diet, the type of snacks participants consumed, the consumption of sweets or cakes as well as fruits and vegetables, having or skipping breakfast, and water intake.

#### Confidence in Diabetes Management

Participants answered 3 questions focused on their confidence regarding diabetes management and meal planning before and after using the platform for 8 weeks. These questions (scored from 1=*very unconfident* to 10=*very confident*) were suggested by diabetes experts based on their experience of evaluating changes in people’s confidence in managing diabetes.

#### System Usability

The System Usability Scale assessed the platform’s usability [23]. This instrument has good psychometric properties and uses a 5-point Likert scale ranging from 1=*strongly disagree* to 5=*strongly agree* across 10 items. A total score of ≥68 is considered *above-average usability* [24].

#### Expectations and Satisfaction

Before they accessed the platform, participants answered 4 questions (scored from 1 to 10) on their expectations of using it, with a score of 1 representing *very strongly disagree* and a score of 10 representing *very strongly agree*. These questions were amended slightly to capture satisfaction with using the platform for 8 weeks. Participants also answered questions on how satisfied they were with individual platform elements as well as whether they learned anything new, encountered any technical issues, and regarded the platform as user-friendly. Participants indicated how motivated they were to use the platform, whether they would recommend the platform to others, whether they would like to keep using the platform, and how the COVID-19 pandemic affected their use of the platform. Finally, they were asked to provide a general rating (ranging from 1 to 10) as well as recommendations on platform improvement.

#### Platform Use

User analytics were collected through Mixpanel software [16] by the platform developer. Data were collected on the number of platform sessions, saved recipes, recipes added to the meal plan, views of the shopping list, and engagement score for each participant who consented for their data to be shared with the research team. On the basis of platform use, the platform developer provided engagement scores classified into 5 groups (very heavy [score: ≥50], heavy [score: 20-49], medium [score: 10-19], light [score: 1-9], and none [score: 0]).

#### Semistructured Interviews

Semistructured interviews were conducted with platform users (7/23, 30%) and diabetes experts (n=3) to gain an understanding of platform usability and potential efficacy. Participants were interviewed individually over the web (via Microsoft Teams) or via telephone for up to 1.5 hours. Screen sharing was used to ensure understanding between the researcher and the participant when discussing specific aspects of the platform. Multimedia Appendix 3 presents the outline for the semistructured interview with participants, which was slightly amended for the diabetes experts. Audio recordings were transcribed verbatim. Thematic analysis was performed by 2 independent and experienced mixed methods researchers (KB and NH). By combining inductive and deductive coding using the interview outline as an initial coding frame, codes were created that were then clustered into themes and subthemes. Agreement was reached through comparison, discussion, and reflection [25]. Sample size was based on our expectation of reaching data saturation.

### Statistical Analysis

Descriptive statistics were used to describe participants’ general and diabetes-related health indicators before and after using the platform for 8 weeks. To get an indication of the strength of evidence against the null hypothesis of no difference in indicators before and after using the platform for 8 weeks, the Wilcoxon matched pairs signed rank test was performed. In case of missing values, the whole pair was excluded from the analyses. This was supplemented by calculation of the Cliff δ effect size, which represents the probability of the superiority of 1 variable over the other, that is, the probability that a randomly selected observation from 1 group is larger than a randomly selected observation from another group, minus the reverse probability. The Cliff δ effect size ranges from −1 to 1, with 0 indicating stochastic equality of the 2 groups, where 1 indicates that 1 group shows complete stochastic dominance over the other group, and a value of −1 indicates the complete stochastic domination of the other group [26]. The values of 0.15, 0.33, and 0.47 corresponded to small, medium, and large effect sizes, respectively [27]. Descriptive data analyses were conducted using SPSS software (version 26.0; IBM Corp) [28], whereas inferential data analyses were conducted using R Core Team [29] software (R Foundation for Statistical Computing).

## Results

### Baseline Characteristics

**Figure 4:**
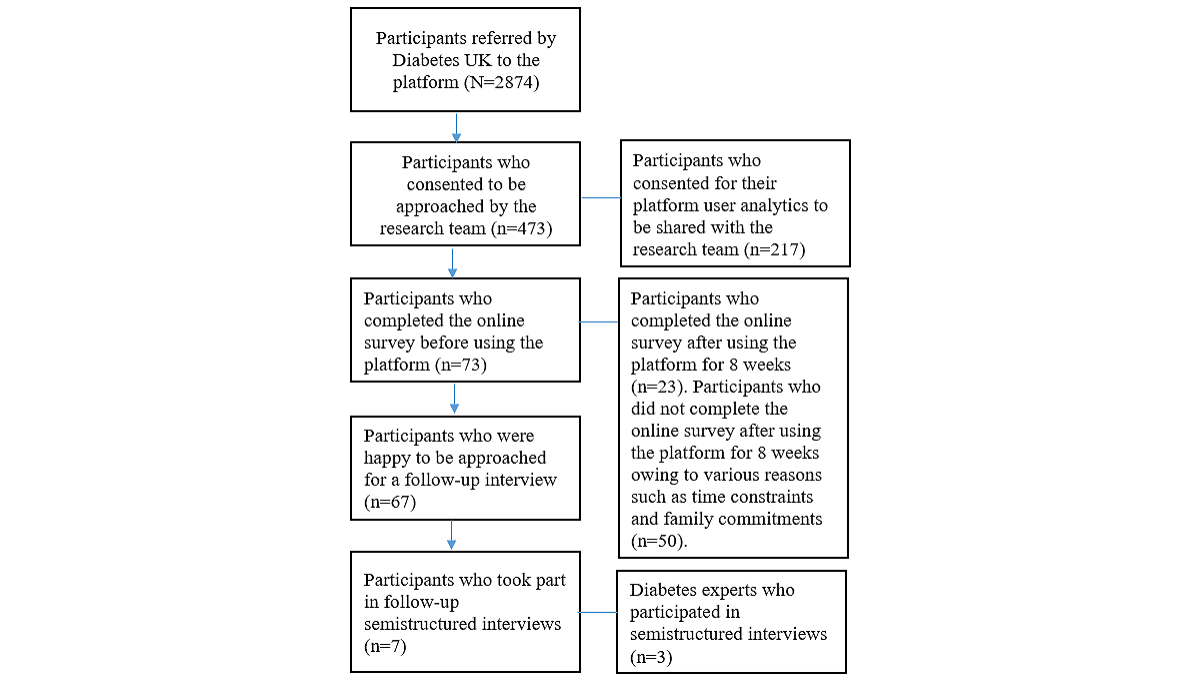
Recruitment and study flowchart of platform users.

Participants’ sociodemographic characteristics at study start are presented in Table 1, and the recruitment flow is presented in Figure 4. Of the 73 participants who completed the baseline web-based survey, 23 (32%) filled in the survey after using the platform for 8 weeks. Of these 23 participants, 17 (74%) filled in the survey completely, 5 (22%) filled in >70%, and 1 (4%) completed 57% of the survey.

The survey participants were from England (61/73, 84%), Scotland (8/73, 11%), and Wales (4/73, 5%). More than half (42/73, 58%) of the participants had a diagnosis of type 2 diabetes. The average age of the participants was 59 (SD 11.1) years, with the youngest participant being aged 27 years and the oldest participant being aged 79 years; most of them were semiretired (28/73, 38%). Most of the participants were of White ethnicity (68/73, 93%), followed by Asian or Asian British (3/73, 4%). In terms of religion, participants stated that they were Christians (44/73, 60%), atheists (21/73, 29%), Hindus (2/73, 3%), Muslims (2/73, 3%), or that they practiced another religion (2/73, 3%). Participants were perceived as highly experienced in their computer use as indicated by a mean total CPQ-12 score of 27.5 (SD 3.5; range 14.5-30.0).

Most of the participants intended to access the platform on their iPhone or iPad (31/73, 43%), computer or laptop computer (21/73, 29%), or Android smartphone or tablet device (21/73, 29%). Most of the participants (33/73, 45%) had data access through Wi-Fi and the mobile phone network, whereas 30% (22/73) of the participants solely relied on Wi-Fi signals, and 25% (18/73) relied on a mobile internet connection. Participants mainly used their device to search for information (27/73, 37%), make calls and send SMS text messages or electronic messages (24/73, 33%), and use social media (10/73, 14%). Other uses included work, shopping, music, photography, and writing. The participants were already using a wide variety of apps (eg, Weight Watchers, Nutracheck, Noom, MyFitnessPal, and Myzone, FreeStyle Libre Sensor) on their device as well as sensors (eg, FreeStyle Libre Sensor) to monitor nutrition (37/73, 51%), physical activity (27/73, 37%), and blood glucose levels (20/73, 27%).

**Table 1:** Sociodemographic characteristics of survey participants at study start (n=73).

Characteristics			Values
Sex, female, n (%)			58 (79)
Age (years), mean (SD)			59.0 (11.1)
**Diabetes, n (%)**			
	Type 1	10 (14)	
	Type 2	42 (58)	
	Prediabetes	12 (16)	
	Relative with diabetes	9 (12)	
**Employment, n (%)**			
	Full time or part time	29 (40)	
	Unemployed or unable to work	15 (21)	
	Other	28 (38)	
	Prefer not to say	1 (1)	
**Education level, n (%)**			
	University: postgraduate or undergraduate	36 (49)	
	College	23 (32)	
	High school or secondary school	14 (19)	
Marital status: married (civil partnership) or cohabitating, n (%)			54 (74)
**Number of people living in household, n (%)**			
	1	13 (18)	
	2	39 (53)	
	≥3	21 (29)	
Longstanding illness or disability, n (%)			41 (56)
Current smoking status, n (%)			8 (11)
**Computer proficiency skills, mean (SD)**			
	Computer basics^a^	4.8 (0.5)	
	Printing^b^	4.3 (1.1)	
	Communication^b^	4.9 (0.3)	
	Internet	4.7 (0.6)	
	Scheduling^c^	4.4 (1.2)	
	Multimedia^c^	4.3 (1.2)	

^a^n=70.

^b^n=71.

^c^n=72.

### General and Diabetes-Related Health Indicators

There was no difference in participants’ reported general health status (mean difference [MD] −1.7, 95% CI −9.0 to 6.0; *P*=.61; Cliff δ=−0.05) before and after using the platform. However, weight (MD 4.5 kg/m^2^, 95% CI 1.0-12.0; *P*=.009; Cliff δ=0.33) and waist size (MD 3.9 cm, 95% CI 2.0-6.5; *P*=.008; Cliff δ=0.48) were lower after 8 weeks of using the platform compared with baseline assessments. Most of the participants measured the diabetes-related indicators by themselves, but in some cases, either a medical professional or a nonprofessional else did so. Descriptive statistics of general and diabetes-related health indicators are reported in Table 2.

**Table 2:** Descriptive statistics of general and diabetes-related health indicators before and after using the platform for 8 weeks.

Health indicator	Before platform use (n=73)			After 8 weeks of platform use (n=23)	
	Value, n (%)	Value, mean (SD)	Value, n (%)		Value, mean (SD)
General health (scale 0-100)	72 (99)	66.1 (20.0)	23 (100)		67.6 (21.7)
Height (m)	70 (96)	1.7 (0.1)	N/A^a^		N/A
Weight (kg)	70 (96)	91.0 (22.6)	23 (100)		89.5 (25.6)
BMI (kg/m²)	70 (96)	27.2 (6.7)	23 (100)		32.7 (9.3)
Waist size (cm)	57 (78)	101.0 (17.0)	18 (78)		94.4 (16.4)
Blood glucose level (HbA_1c_^b^, mmol/mol)^c^	44 (60)	37.2 (31.1)	13 (57)		32.9 (27.0)
Blood pressure, systolic (mm Hg)	36 (49)	132.3 (13.7)	9 (39)		122.2 (7.6)
Blood pressure, diastolic (mm Hg)	35 (48)	78.2 (9.5)	9 (39)		75.9 (5.9)
HDL^d^ cholesterol level (mmol/L)^e^	7 (10)	3.5 (1.7)	—^f^		—
Total cholesterol level (mmol/L)	15 (21)	4.9 (0.7)	—		—

^a^N/A: not applicable (height was not measured after 8 weeks of using the platform, given that this is a stable trait).

^b^HbA_1c_: glycated hemoglobin.

^c^Median 42.0 (IQR 7.6-50.75).

^d^HDL: high-density lipoprotein.

^e^Median 3.6 (IQR 1.57-5.10).

^f^HDL and total cholesterol levels are not reported after 8 weeks of using the platform, given that only 2 (9%) of the 23 participants reported these.

### Healthy Eating

Dietary habits before and after using the platform for 8 weeks were comparable (Table 3).

**Table 3:** Descriptive statistics on aspects of healthy eating before and after using the platform for 8 weeks.

Variables		Before platform use (n=73), n (%)	After 8 weeks of platform use (n=22), n (%)
**Participants’ diet is...**			
	Different every day	53 (73)	17 (77)
	Different only sometimes during the week	11 (15)	4 (18)
	Different during weekend days	2 (3)	1 (5)
	Very monotonous	7 (9)	0 (0)
**Snacking habits**			
	I snack	54 (74)	15 (68)
	I do not snack	16 (22)	7 (32)
	Prefer not to answer	3 (4)	0 (0)
**Having breakfast**			
	Always	51 (70)	18 (82)
	Often	9 (12)	1 (5)
	Sometimes	8 (11)	1 (5)
	Never	5 (7)	2 (9)
**Consumption of sweets or cakes (number of times per week)**			
	Never	12 (16)	2 (9)
	Less than once a day	47 (64)	19 (86)
	At least once a day	14 (19)	1 (5)
**Regularity of eating at least 2 portions (200 g) of fruit a day**			
	Always	26 (36)	9 (41)
	Often	27 (37)	10 (46)
	Sometimes	18 (25)	3 (14)
	Never	2 (3)	0 (0)
**Regularity of eating at least 2 portions (200 g) of vegetables a day**			
	Always	33 (45)	12 (55)
	Often	25 (34)	9 (41)
	Sometimes	13 (18)	1 (5)
	Never	2 (3)	0 (0)
**Regularity of drinking at least 1 L of water a day**			
	Always	15 (21)	8 (36)
	Often	25 (34)	3 (14)
	Sometimes	22 (30)	8 (36)
	Never	11 (15)	3 (14)

### Confidence in Diabetes Self-Management

After using the platform for 8 weeks, participants felt most confident in meal planning (mean 6.0, SD 2.6; range 1-10) and making healthy food choices (mean 5.7, SD 2.6; range 1-10). They were least confident about their diabetes management before (mean 5.2, SD 2.6; range 1-10) and after using the platform (mean 5.4, SD 2.6; range 1-10).

### System Usability

After using the platform for 8 weeks, participants reported a mean System Usability Scale index of 50.7 (SD 18.2; range 10-85), which indicated a below-average usability score. More than half of the participants (11/21, 52%) thought that they would not need assistance with using the platform. However, 43% (9/21) found the platform cumbersome to use, and 33% (7/21) found it unnecessarily complex. Of the 21 participants, 10 (48%) indicated that they would like to use the platform more frequently. Table 4 presents participants’ responses in more detail.

**Table 4:** System Usability Scale questionnaire scores for the platform (n=21).

Statements	Disagree^a^, n (%)	Neutral, n (%)	Agree^b^, n (%)
I think that I would like to use the platform frequently	7 (33)	4 (19)	10 (48)
I found the platform unnecessarily complex	8 (38)	6 (29)	7 (33)
I thought the platform was easy to use	7 (33)	6 (29)	8 (38)
I think that I would need assistance to be able to use the platform	11 (52)	6 (29)	4 (19)
I found the various functions in the platform were well integrated	5 (24)	11 (52)	5 (24)
I thought there was too much inconsistency in the platform	6 (29)	13 (62)	2 (10)
I would imagine that most people would learn to use the platform very quickly	4 (19)	9 (43)	8 (38)
I found the platform very cumbersome to use	6 (29)	6 (29)	9 (43)
I felt very confident using the platform	7 (33)	8 (38)	6 (29)
I needed to learn a lot of things before I could get going with the platform	6 (29)	9 (43)	6 (29)

^a^Scores 1 and 2 were combined and clustered under the heading of *Disagree*.

^b^Scores 4 and 5 were combined and clustered under the heading of *Agree*.

### Expectations and Satisfaction

Participants expected that the platform would support them primarily in making healthy food choices (mean 6.1, SD 1.7; range 1-10), planning meals more efficiently (mean 6.0, SD 1.9; range 1-10), diabetes management (mean 5.9, SD 1.7; range 2-10), and their food shopping experiences (mean 5.6, SD 2.1; range 1-10). After using the platform for 8 weeks, 18 (78%) of the 23 participants reported that the platform primarily supported them in planning meals more efficiently (mean 5.0, SD 2.7; range 1-10) and secondarily in diabetes management (mean 4.8, SD 2.0; range 1-9), making healthy food choices (mean 4.8, SD 2.2; range 1-9), and their food shopping experiences (mean 4.8, SD 2.6; range 1-10).

Most of these participants (17/18, 94%) indicated that they did not learn anything new while using the platform but found it to be easy to use (mean 5.6, SD 3.0; range 1-10). Of the 18 participants, 14 (78%) reported that they did not encounter any technical challenges. Half (9/18, 50%) of the participants indicated that they would recommend the platform to other people who have diabetes or are taking care of someone with diabetes. Participants scored 4.7 (SD 2.7; range 1-10) on the question regarding how motivated they were to use the platform, whereas 8 (44%) of the 18 participants said that they would not continue using the platform. The overall average rating of the platform was 5.2 (SD 3.2; range 1-10), and most of the participants (11/18, 61%) thought that the COVID-19–related restrictions did not affect the optimal use of the platform.

### Platform User Statistics

Saving recipes was the most used feature across the platform, followed by adding recipes to the meal plan and viewing the shopping list. Survey participants who consented for their platform data to be shared and used the platform actively represented very heavy (3/33, 9%), heavy (5/33, 15%), medium (5/33, 15%), light (13/33, 39%), and none (7/33, 21%) categories of users (Tables 5 and 6).

**Table 5:** Platform user analytics per user group across total and survey sample (n=217).

User pattern	Saved recipes, n^a^	Frequency of viewing the shopping list, n	App sessions, n	Recipes added to the meal plan, n
Very heavy	663	50	313	91
Heavy	425	20	69	99
Medium	173	13	40	40
Light	132	33	15	21
None	0	0	0	0

^a^The total amount of times 217 people saved a recipe.

**Table 6:** Platform user analytics across a subsample of survey participants (n=33).

Feature	Values, n^a^	Value, median (IQR; range)
Saved recipes	18	5.5 (2-17.25; 1-110)
Frequency of viewing the shopping list	13	1.0 (1-3; 1-13)
App sessions	6	6.5 (2.50-44.25; 1-144)
Recipes added to meal plan	9	3.0 (1-7.50; 1-14)

^a^The total amount of times people who completed the survey (n=33) saved a recipe.

### Semistructured Interviews

#### Overview

Semistructured interviews were conducted during the COVID-19 pandemic. There was a general trend of people cooking at home more and trying out web-based recipes. The meal planner and web-based food shopping features became more popular during the COVID-19 pandemic because people wanted to restrict their outside shopping trips, and this held true regardless of low product stock and limited availability of delivery slots.

#### Theme 1: Usability

Participants described minor usability issues while using the platform. They indicated that the filter option for finding specific recipe content was not very visible:

[S]o I know that here you’ve got a filter system, but I think that’s not very obvious.

Diabetes expert 1

The layout of the *diabetes-friendly* community page on the platform was perceived as unclear. According to participants, there was an information overload on the home page and across platform features; in addition, measurement units, spelling, and ingredients were American and should be British:

I very rarely use a cup as a measurement because I don’t know what it is, I don’t know how big their cup is and this one has got yeast, so it’s important that you have got the right quantity of flour to yeast.

Platform user 1

Some of the participants found the recipe titles and cooking instructions on the platform (specifically the *diabetes-friendly* community page) unclear:

I would say most of the recipes that I’ve clicked on were quite good, but I saw this recipe for gnocchi, and I thought, oh yes, I quite like that, I’ll make that. And the instructions just didn’t make sense, total rubbish. I thought, there’s something gone wrong when they included that recipe in there...But yeah, I think you’ve got to check the recipes and make sure they’re at least readable, and understandable.

Platform user 2

Furthermore, information about portion size per serving was not displayed, it was not possible to change serving sizes for all imported third-party recipes, and there were synchronization issues among different devices and even among different users sharing and using the same web-based shopping list.

Participants perceived the platform as intuitive, user-friendly, and easy to use, mainly because it was primarily image based, and information was consistently and clearly presented across the platform:

I think because it’s so image based, it just makes it more engaging and easier to click, so I do think that helps. I know that’s not really...well, it’s part of the user experience, but I found it very easy to use generally, I’m just more about the...it’s easy to click and easy to add and all these things that I don’t think are terribly hard to use.

Diabetes expert 1

Participants felt that there were sufficient instructions across the different platform features, which were easy to follow, although a platform user would have preferred more instructions:

Platform features were easy enough to use...yeah, even for somebody like me who is not a computer expert.

Platform user 2

It took a few months before it was user friendly for me because it took me a while to understand it and I had to ask my daughter for some advice and she was able to do that. She said if you just press home and you go from there again, start again...and find the recipe I wanted, and press on it again.

Platform user 3

Participants reported that navigation was easy and intuitive on their smartphones, but they preferred to use a larger screen (eg, tablet device or laptop computer) or printed recipes to follow instructions while cooking. A diabetes expert suggested that it might be easier to use the meal planner on a desktop computer because of the *drag-and-drop* functionality.

#### Theme 2: Perceived Usefulness

Participants perceived the platform as a useful starting point for people with diabetes. Others mentioned its usefulness for people with other health conditions, those who are less experienced cooks, and those who would like to eat healthier. The diabetes experts appreciated that the platform is community driven, and the indicators of popularity (eg, number of recipe likes and community members) were seen as useful by some. Participants reported that the platform offered a great deal of recipe inspiration and enabled people to conveniently collect and save recipes in 1 place.

Most of the diabetes experts were advocates of communicating glycemic index and load information, whereas the platform users mainly appreciated nutritional information such as carbohydrates, fat, and calories per serving. The diabetes experts thought that the meal planner was useful in offering structure as well as an overview of ingredients to support people in preparing a variety of meals throughout the week:

I think that’s all kind of useful stuff to help people meal plan better so that they’re not kind of trying to have new things every single day and that kind of recognition that you can repeat things throughout the week, that type of thing.

Diabetes expert 2

They also thought that the web-based shopping feature was convenient and could save time even when the web-based shopping list was used to shop for items in a physical supermarket:

I just think the fact you can transfer to your shopping list makes it easier and on the go as well. People take their phone with them shopping, don’t they?...It’s great to see how it can easily be added to your shopping list in stores...That is a big asset, transferring it because it’s very...Recipes and writing a shopping list, it’s time consuming.

Diabetes expert 3

Since like the New Year we’ve kind of did a big push to eat a bit better and also we found the shopping list and kind of meal planner bit really, really useful...I don’t think we’d kind of fully explored how we could integrate the planner and the shopping lists, that kind of element was quite a game changer on our part.

Platform user 4

However, the platform users did not use the meal planner and shopping list features frequently, and some of them preferred paper-and-pencil methods because they perceived them to be a more flexible approach for people who are not very smartphone oriented:

At the moment I would say personally, no, but that’s because as I’ve explained that, you know, that it’s easier with pen paper, but I could see the facility would be useful for some people. I don’t knock the facility for those who work in that way and are very much attached to their smartphone, then I could see it being really useful tool.

Platform user 5

Participants did not involve family members in their journey of using the platform but would definitely share it in case they thought it would be beneficial for them. However, some of the participants stressed the potential and enjoyment of sharing the platform with family members:

So me and my partner both have the app now, we share like a shopping list on there with each other and...Normally I will cook my own lunch through the app and then we’ll kind of cook a dinner together, normally from a recipe that we’ve both found on the app or that we’ve imported from erm...you know, from a website or from a cookery book.

Platform user 4

#### Theme 3: User Experiences

Participants perceived the platform as attractive—nice, clean, fresh, and simple—but did not use it frequently. It provided a good variety of features, including the possibility to search, save, and like recipe content across different communities. The platform users expressed that the recipes were appetizing and seem to offer healthy food options for people with diabetes:

I liked the recipes because I hadn’t any idea what to cook for somebody that’s prediabetes, it’s something new to us, so I was very keen to see.

Platform user 3

The drag-and-drop functionality used to transfer ingredients from the meal planner to the shopping list as well as the option to share the shopping list with relevant others were highly appreciated by some. In addition, some of the platform users indicated that the platform helped them to prevent food waste because they selected recipes on the basis of ingredients available at home.

#### Theme 4: Health Advice

**Figure 5:**
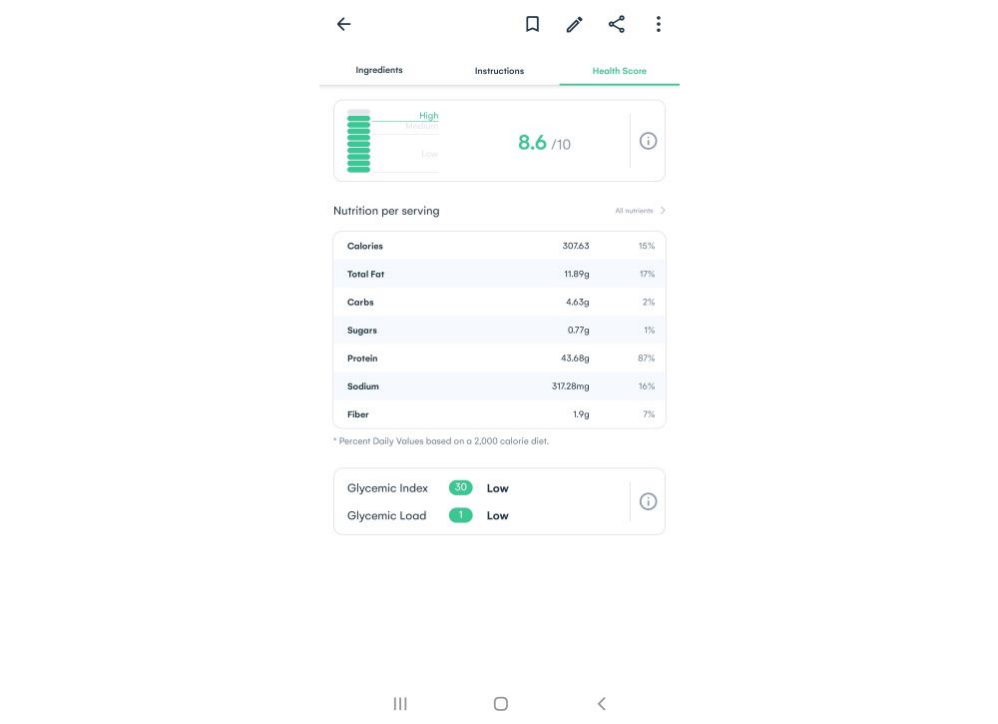
Screenshot of the platform showing the health score and nutrition per serving.

Participants reported that the health score (Figure 5) displayed by the platform did not align with the traffic light labeling system and nutritional recommendations used in the United Kingdom.

According to the diabetes experts, it needs to be clear how the US-based health score was calculated and interpreted before presenting it to people with diabetes:

I felt that some of the recipes, the nutrient score, the health score didn’t necessarily reflect how healthy that recipe actually was. So I don’t know if that’s because of the nutrients that have been used. So like here, for example, this recipe gets a score of 5.9 and to me the only thing really that would be a negative is the salt. So yeah, I just wondered what the kind of justification was behind having all carbohydrates being a negative impact. I know they’ve got the fiber here as a positive so...

Diabetes expert 2

One platform user with type 1 diabetes noticed that there was a difference between the nutritional values (specifically with regard to carbohydrates) displayed within the platform and those presented with the original recipes. A diabetes expert pointed out the potential risk of different nutritional analyses when American cups are widely used for measurement in British households. It is important to present information accurately and clearly because this can have direct implications on people’s blood glucose levels. The diabetes experts also thought that healthy recipe content review and approval by dietitians were necessary to ensure suitability for people with diabetes:

[P]eople post anything on there that’s not vetted. I’ve got to be honest. That is a concern. It’s great it’s got choice and if you’ve got the knowledge, it is useful, but then, I’ve only spent 40 minutes with you and I’ve already clicked on quite a few things where we wouldn’t have it on our website. People with diabetes, both type 1 and type 2 are increased risk of cardiovascular disease and you’ve got a recipe with pure saturated fat. Of course I’m going to be concerned.

Diabetes expert 3

Participants stressed the importance of not interpreting the platform as giving individual (medical) advice on what to eat, particularly when it has not been checked by a health care professional.

#### Theme 5: Potential to Improve Diabetes Management

The diabetes experts reported that labels such as *diabetes friendly* and *low carb* might be confusing for people with diabetes because a suitable diet is very individualized. They thought that it would be more important to focus on portion size, nutritional value, and type of carbohydrates. The majority of the participants felt that the platform offers a great variety of recipe inspiration and support for people with diabetes in meal planning and healthy eating habits:

People who are maybe looking for starting on that “how do I start to eat healthily,” it’s a good starting point, I would say definitely, and there’s a lot of recipes that means you could go with this for quite a number of weeks...I think most of the recipes that I’ve looked at are relatively straightforward. So, I don’t think they’re too overwhelming for people if suddenly they think how I am going to go with this.

Platform user 5

So in terms of the planner and the shopping I think the planner itself will just kind of help people to...I mean it has the potential to help people kind of be thinking more proactively about what they’re eating rather than just making decisions on the spot or last-minute kind of decisions or whatever so it gives you that kind of forward thinking so that perhaps you can then decide about a range of different, you know...it potentially could help people to make healthier choices.

Diabetes expert 2

Participants agreed that some education about what healthy eating entails and increasing knowledge of diabetes management were needed. The participants who were already quite knowledgeable about their diabetes and the role of nutrition felt that the platform did not contribute much to their diabetes management; however, they thought that it could be useful for those who have been recently diagnosed:

Again, I think the reason primarily for not is because I’ve lived with it for quite a long time, so a lot of that information is already in my head, but again going back to people who are newly diagnosed and I’d say in that, really thinking in terms of it from diagnosis and the first 12 months are the time when people feel that they’re under pressure, and so that system I feel probably again would be very helpful for people in that situation.

Platform user 6

Some of the participants mentioned that the platform will only support diabetes management if people commit to changing their behavior and sustaining healthy habits, as well as have a good level of understanding about how food affects diabetes-related health indicators.

#### Theme 6: End-User Diversity

Participants reported that the platform had a universal approach suitable for anyone who wants to try out different recipes but would naturally attract people who speak English and are able to understand and use modern technologies. Because of the platform’s ease of use, this can include younger as well as older people, with the latter normally less experienced in using modern digital technologies. The diabetes experts mentioned that the platform has potential to involve the wider support system of people with diabetes (eg, friends, carers, and family members).

Participants mentioned that the platform displays some recipes from international cuisines (such as Asian and Indian but not African or Chinese), but it would not appeal to Asian, Black, and minority ethnic groups because it does not offer enough recipe variety:

It does strike me, and it may be that the people that are submitting the recipes are very city-centric, a bit London, affluent, digitally aware, disposable income...I don’t think it would, in the current format, appeal to a broad range of ethnicities or demographics. Some of the ingredients, for example. You’re looking at them. Elderflower syrup is on this one.

Diabetes expert 3

[W]hether it’s getting people from all ethnicities to join, I don’t know. Plus obviously, it’s all in English.

Platform user 1

Some of the participants indicated that the platform can never be fully inclusive but that this should be accepted because it offers enough variety and options for those who are interested. A diabetes expert indicated that feeling part of a community is more complex than sharing recipes over the web. Participants indicated that displaying a budget across recipes and including budget supermarket chains would potentially be helpful to reach communities with a lower socioeconomic status, specifically during the COVID-19 pandemic with more people being on furlough. Visual impairments and learning disabilities are quite common in people with diabetes; therefore, improving accessibility needs to be further considered.

#### Theme 7: Comparison With Other Apps and Platforms

Although there are other healthy recipe websites and applications available, participants reported that this platform is unique in offering a multicomponent approach by offering individual recipes, meal planning, and web-based shopping on 1 platform to support people with diabetes. The diabetes experts mentioned websites supporting education on diabetes and dieting and providing exercise applications that display nutritional analyses based on inserted data as well as applications to support counting carbohydrates and calories. Some dieting applications provide recipe videos in addition to written content, thereby making them accessible to a broader audience.

#### Theme 8: Recommendations for Improvement

Participants proposed several platform improvements regarding recipes, portion sizes, education and management, budget, peer support, tailored content, and layout (Multimedia Appendix 4).

## Discussion

### Principal Findings

This mixed methods study aimed to examine the usability and preliminary efficacy of an AI-driven web-based nutrition platform to support diabetes management. The survey and semistructured interview results showed that the platform was well received and primarily supported people with diabetes and their carers in identifying healthy recipes but less so in supporting meal planning and creating web-based food shopping lists. Although the diabetes-related health of most of the participants was largely stable, the platform was seen as attractive and a good starting point for recipe inspiration. The weight and waist circumference of participants tended to decrease after using the platform. However, because this is a small before-and-after study without a control group, it is not possible to conclude whether the improvements can be explained by actual platform use or other factors; therefore, these results should be interpreted with caution. High-quality robust trials are needed to examine its effectiveness on general and diabetes-related health outcomes.

Although recruitment was national, participation in the web-based surveys and semistructured interviews was low. This could be due to the COVID-19–related lockdown being associated with poorer mental health, especially among young women from ethnic minority groups who felt lonely and experienced prepandemic illness [30]. Therefore, they may not have prioritized research participation. Our sample seemed biased toward participants who were more willing to participate in study procedures [31], including primarily participants who are women, older, diagnosed with type 2 diabetes, nonsmoking, tech savvy, from a 2-person household, and of White British ethnic background. However, based on the semistructured interview results, participants expected that people who were less proficient with technology would not encounter any issues in using the platform because it is largely image based, easy to use and navigate, and only suffered from minor usability issues (eg, synchronization, American spelling, and measurements). Although the platform has been developed in line with accessibility and readability guidelines [32], the fact that participants preferred a larger screen to read instructions from while cooking and wrote down their shopping list using paper and pencil could mean that it was not experienced to be as accessible as it could be, considering potential vulnerabilities across the population with diabetes [33,34]. The diabetes experts recommended that visual impairments, ethnicity, and socioeconomic status should be considered when designing digital applications for patients with diabetes. Future applications should design readability and accessibility features that can be tailored to individual preferences, thereby increasing overall user experience [35].

In addition, participants reported a relatively high general health status and largely healthy eating habits, and their diabetes-related health indicators seemed to be within the normal range, apart from some of them being overweight (18/70, 26%) or having slightly elevated HDL cholesterol levels (22/70, 31%). Participants felt relatively confident about their ability to make healthy food choices, plan meals efficiently, and manage their diabetes. This could partly explain why participants were only moderately motivated to use the platform and did not use the platform on a regular basis, as demonstrated by the platform user analytics.

On the basis of the semistructured interviews, participants indicated that the platform might be more relevant for people who have difficulties with managing their diabetes and are not aware of the impact of their eating habits (including nutritional values), had been recently diagnosed, or were on a waiting list to see a dietitian during the COVID-19 pandemic. The diabetes experts also felt that the platform would be beneficial for people with other long-term health conditions and people aiming for a planned approach to healthy nutrition and weight loss, which seems important in preventing diabetes. They were appreciative of the platform’s potential but also foresaw challenges in user-posted recipes as well as the accuracy of nutritional information and therefore felt that moderation by dietitians is needed. Indeed, this concern has also been raised across other web-based platforms providing specific nutritional information [36]. The diabetes experts also questioned whether the web-based platform would be able to realize actual behavior change over time. On the basis of a systematic review of web-based self-management programs for people with type 2 diabetes, it was clear that most of the studies (8/13, 62%) did not include a long-term follow-up [37]. Across the 5 studies that included follow-up assessments, only 1 (20%) assessed health outcomes beyond a 1-year follow-up. Future efficacy studies should include a longitudinal design to capture long-term effects in diabetes-related and general health outcomes and see whether improved lifestyle behaviors persist over time.

Despite our study not being representative of the population with diabetes and being too small to perform any kind of subgroup analyses (participants with high motivation vs those with low motivation), it still contributes to well-perceived platform experiences and its potential to support diabetes management. A variety of recruitment strategies, including using social media platforms to attract a younger population [38], should be explored in future studies to increase diversity in engagement and participation. This includes Asian, Black, and minority ethnic communities as a group with a high risk for diabetes [39,40], while also considering those with lower social economic status and comorbidities such as visual impairment [33,34].

Participants expected moderate levels of contribution of the platform toward making healthy food choices, planning meals efficiently, and managing diabetes. Indeed, the satisfaction scores indicated that the additional contribution of the platform regarding these aspects was below average, except for efficient meal planning. Although the meal planner was not frequently used, it could be that the use of recipes offered structure for cooking and meal preparation. High levels of self-efficacy are positively related to diabetes self-management behaviors [41], and this is likely to be the case in our study, given that the diabetes-related health indicators were mostly stable. Further research with larger samples will need to examine the role of motivation and self-efficacy in web-based interventions aimed at improving diabetes management [42].

### Limitations

As participants were relatively healthy, and response rates were low, our sample and results may not adequately reflect the community of people with diabetes or only represent a small part of the community, namely participants who are healthier and may be more willing to cooperate or contribute to intervention research and its procedures. The reasons for high attrition on the survey could be explained by limited financial incentives, the absence of a prenotification to potential participants, and the use of a fully web-based survey instead of sending over the web as well as paper-based surveys [43].

Another reason could be that participants who did not use the platform on a regular basis or only used 1 aspect (eg, saving recipes) of the platform felt that they would not add value by filling in the survey about the platform, although we tried to preempt this by stating that regardless of how many times participants used the platform, completing the survey would still provide us with relevant insights into how to improve the platform. On the basis of the semistructured interviews, it seems that participants indeed showed curiosity in the beginning but did not maintain their interest over time, especially in the case of people who managed their diabetes relatively well and therefore felt that the platform was less relevant for them. The same pattern of engagement is seen in other web-based applications for chronic illnesses [44]. Although the reported usability issues were limited, high levels of engagement over a longer period of time with a web-based platform and study procedures remains challenging, and more research is needed in this area to determine which factors contribute to increased engagement, specifically for people with diabetes. Future research should anticipate higher attrition rates across participants who are chronically ill as well as older adult participants and account for this in their recruitment targets [45,46] while also considering different preferences in terms of delivery mode among participants to improve survey response rates.

In addition, Asian, Black, and minority ethnic communities, who normally display a higher prevalence rate of type 2 diabetes than European communities, were underrepresented in our study [39,40]. Therefore, as in the majority of other studies, our results cannot be generalized to the wider community of people with diabetes and need to be interpreted with caution owing to the study’s small and potentially biased sample. Robust and sample-diverse studies are needed to help inform subsequent priorities of research and applications in this area and draw conclusions more reliably across the whole community of people with diabetes.

### Comparison With Prior Work

On the basis of platform user statistics, most of the participants (151/217, 69.6%) did not use the platform on a regular basis. This pattern is seen regularly across mHealth apps for diabetes where support tools are positively received, but the actual use is relatively low [47]. People who are managing diabetes using mHealth apps often need a reminder or push notification [47], which is not a default setting in this platform. This may need to be considered when the platform is offered to people with chronic health conditions to increase their engagement and motivation to use features for recipes, meal planning, and creating web-based shopping lists. Apart from personalized recipe suggestions, using AI could improve engagement with such platforms and diabetes care [48]. Although participants were experienced in terms of computer use, and the majority (42/73, 58%) owned a smartphone or laptop computer as demonstrated by the survey, it seemed from the semistructured interviews that participants did not embrace technology fully in daily life, as illustrated by some of the interview participants not taking their mobile phone with them while shopping or their preference to write down their shopping list using paper and pencil as well as to write down their favorite recipes in a notebook. This differentiates them from younger *digital natives* (defined as being born *digital* and therefore born in or after 1980 [49]) who share and synchronize their recipes and meal plans with relevant others, as demonstrated by an interview participant. However, this pattern contradicts the findings from a European cross-sectional survey study in which digital natives with type 1 diabetes used information and communication technology daily but not to support their diabetes care [50]. More research is needed on the barriers and facilitators to the use of technology across different digital age–specific groups.

Survey participants gave a relatively poor (or *OK*) usability rating to the platform, whereas the results from the interviews indicated that participants only experienced minor usability issues and felt that the platform was easy to use, user-friendly, intuitive, and contained clear instructions. The platform was mainly seen and used as a recipe inspiration platform in which recipe suggestions were prompted based on deep learning algorithms. The interview participants suggested that the platform would be useful as a starting point specifically for people recently diagnosed with diabetes (or other long-term chronic conditions) to eat healthier. This is particularly relevant, given the long waiting list of patients to be seen by dietitians or health care professionals and limited accessibility specifically during the COVID-19–related lockdowns.

Semistructured interviews with people with diabetes, their carers, and diabetes experts resulted in some recommendations to improve the platform. These mainly focused on recipes, administrator rights, portion size, education, budget, peer support and tailored content, and layout. An estimation of the cost per recipe will be useful for people affected by the current cost-of-living crisis and specifically support people from deprived areas where type 2 diabetes is more prevalent [34]. According to the diabetes experts and some of the platform users, it is important that nutritional information is validated and in line with the UK traffic light labeling system. Presenting nutritional information on calories and carbohydrates seems specifically relevant for people with type 1 diabetes, however, imprecise display of this information was seen as a potential barrier by an interview participant and the diabetes experts, especially given that other mHealth apps are available that display and calculate nutritional information more accurately and reliably [51]. Most of the participants acknowledged the importance of some education on the role of nutrition and physical activity levels in diabetes management, especially for people who had been recently diagnosed or lacked knowledge, before accessing the platform. This seems fruitful because knowledge, attitude, and practice seem to be positively related to glycemic control [52]. Although studies have suggested that education is an important aspect to improve existing mHealth apps in diabetes management, this has only been adopted by a few apps [15,47]. As the educational component was provided primarily in the nutritional values displayed in user-posted recipes, it is recommended to modify the web-based platform, which could be cocreated by people with diabetes and health care professionals, thereby using existing learning resources on diabetes management and minimizing costs [53].

On the basis of the diabetes experts’ input during the semistructured interviews, it should be noted that this platform does not in any way provide health or medical advice because nutrition in the context of diabetes is very individualized, and nutrition advice should always be sought from an appropriate health care professional. It was recommended to include some dietary monitoring of the recipes that are posted and shared across the community to ensure that all of them are in agreement with the UK national food-based dietary guidelines and traffic light labeling system [54]. This should be taken on board in further applications of the platform in the context of the management of long-term health conditions, such as diabetes.

### Conclusions

The AI-driven, web-based nutrition tool was perceived as accessible and easy to use, with minimal usability issues. Several important recommendations for its improvement have been made, and the relevance of education on healthy eating for specific groups has been stressed. Most of our participants were quite knowledgeable and stable regarding the self-management of their diabetes. The potential of the platform’s meal planner and shopping list was acknowledged by the diabetes experts, but participants thought it was mainly useful for recipe inspiration. Diabetes experts indicated that the recipe content should be reviewed by experts to enable people with diabetes to maintain a reliable and healthy personalized diet.

Given that Asian, Black, and minority ethnic communities and other groups considered susceptible were underrepresented in this study, future research should deploy different recruitment strategies to involve a more representative sample of people with diabetes who could potentially benefit from this platform. This includes making a distinction among digital age–specific groups. Future applications should consider tailored accessibility and readability features to increase overall user experience. Although some reductions in weight and waist circumference were found, no causal inferences can be made because of the small sample size and the study’s pretest-posttest design. Longitudinal studies should examine the efficacy of web-based platforms regarding diabetes-related and general health outcomes. Cocreating solutions with people with diabetes and health care professionals and further development of AI technology have great potential to improve diabetes management in a more engaging and personalized manner. Motivation and self-efficacy are expected to play an important role, and theoretical underpinnings should be considered in intervention development and future studies.

## Appendix

Multimedia Appendix 1 Reporting checklist for this cross-sectional study.

Multimedia Appendix 2 Original protocol for the study.

Multimedia Appendix 3 Outline for semistructured interview with participants.

Multimedia Appendix 4 Participants’ recommendations for platform improvements.

## Abbreviations

AI: artificial intelligence
CPQ-12: Computer Proficiency Questionnaire-12
EQ-VAS: EuroQol Visual Analog Scale
HbA_1c_: glycated hemoglobin
HDL: high-density lipoprotein
MD: mean difference
mHealth: mobile health
STROBE: Strengthening the Reporting of Observational Studies in Epidemiology

## Data Availability

The data set is available from the Figshare repository [55].
