# Supplementary material for: Usability and Preliminary Efficacy of an Artificial Intelligence–Driven Platform Supporting Dietary Management in Diabetes: Mixed Methods Study
Source: JMIR Hum Factors. 2023 Aug 9;10:e43959. doi: 10.2196/43959 (PMC10448291; doi:10.2196/43959)
Supplement: Multimedia Appendix 2 [file humanfactors_v10i1e43959_app2.docx]

**Platform evaluation**

**PROTOCOL**

| **Funder** | West Midlands Academic Health Science Network (WMAHSN; hosted by Birmingham NHS Foundation Trust) |
| --- | --- |
| **CU ethics reference** | P109725 |
| **CU ethics approval date** | 26/08/2020 |
| **Version number** | 1.0 |
| **Date** | 12-08-2020 |
| **Start date** | 28/07/2020 |
| **End date** | 28/07/2021 |

**KEY PROJECT CONTACTS**

| **Project Leader** | Dr. Kim Bul  Coventry University  Centre for Intelligent Healthcare  [kim.bul@coventry.ac.uk](mailto:kim.bul@coventry.ac.uk) |
| --- | --- |
| **Other project members** | Nikki Holliday  Coventry University  Centre for Intelligent Healthcare  [nikki.holliday@coventry.ac.uk](mailto:nikki.holliday@coventry.ac.uk)  07557425667  Rachael Molitor  Coventry University  Centre for Intelligent Healthcare  [ac3486@coventry.ac.uk](mailto:ac3486@coventry.ac.uk) |
| **Funder** | WMAHSN  [logan.ryan@wmahsn.org](mailto:logan.ryan@wmahsn.org)  07920580061 |

**PROJECT SUMMARY**

| **Full project title** | Platform evaluation | |
| --- | --- | --- |
| **Project aim** | The aim is to assess the impact of the online nutrition platform, distributed through Diabetes UK, on important health and intervention outcomes. | |
| **Project design** | Mixed method approach using descriptive and inferential (where possible) statistics on platform data and surveys. Thematic analyses will be performed on case studies and semi-structured online interviews. | |
| **Project participants** | Project participants are people with Type 1 or Type 2 Diabetes (or at risk) as referred by Diabetes UK through their website. Other stakeholders such as platform developer and Diabetes UK staff (i.e. dieticians) will be interviewed for implementation purposes and expert opinions. | |
| **Project arms** | Surveys will be distributed to examine several health and intervention outcomes before and after using the platform. | |
| **Sample size** | Sample size of data collected through survey and Mixpanel data is estimated to be (estimated n= 200-300), case studies (n=4), semi-structured online interviews (n=24) including up to 3 dieticians working for Diabetes UK and up to 3 platform developer staff members. | |
| **Planned project period** | 12 months | |
| **Planned recruitment start date** | 01/10/2020 | |
| **Planned recruitment end date** | 01/11/2020 | |
| **Planned project end data** | 28/07/2021 | |
|  | **Objectives** | **Outcome Measures** |
| **Primary** | The primary aim is to assess the impact of the online platform on several health and intervention outcomes | Health and intervention outcomes |

# Key Words: evaluation, diabetes, nutrition, app, mixed method

**SCHEDULE OF OBSERVATIONS**

*Table 1: Schedule of Events*

| Procedure | Screening | Data collection | Intermediate summary report | Summary report |
| --- | --- | --- | --- | --- |
| Recruitment through Diabetes UK website | X |  |  |  |
| Informed consent | X | X |  |  |
| Primary health and intervention outcomes – collected through Mixpanel, case studies, semi-structured interviews and surveys |  | X |  |  |
| Intermediate summary report of project results |  |  | X |  |
| Final summary report of project results |  |  |  | X |

# LIST of CONTENTS

| **GENERAL INFORMATION** | **Page No.** |
| --- | --- |
| TITLE PAGE | 1 |
| KEY PROJECT CONTACTS | 2 |
| PROJECT SUMMARY | 3 |
| SCHEDULE OF OBSERVATIONS | 5 |
| LIST OF CONTENTS | 6 |
| LIST OF ABBREVIATIONS | 8 |
|  |  |
| **SECTION** | |
| 1. INTRODUCTION | 9 |
| 2. RATIONALE | 9 |
| 3. OBJECTIVES & OUTCOME MEASURES/ENDPOINTS | 10 |
| 4. PROJECT DESIGN | 11 |
| 5. PROJECT SETTING | 11 |
| 6. ELIGIBILITY | 12 |
| 7. TRIAL PROCEDURES | 12 |
| 8. STATISTICS & DATA ANALYSIS | 18 |
| 9. DATA MANAGEMENT | 19 |
| 10. TRIAL OVERSIGHT | 20 |
| 11. MONITORING, AUDIT & INSPECTION | 21 |
| 12. ETHICAL AND REGULATORY CONSIDERATIONS | 21 |
| 13. DISSEMINATION POLICY | 21 |
| 14. REFERENCES | 22 |
| 15. Attachment_1: INVITATION EMAIL FIRST SURVEY | 23 |
| 16. Attachment_2: PARTICIPANT INFORMATION SURVEY | 24 |
| 17. Attachment_3: INFORMED CONSENT FORM SURVEY | 26 |
| 18. Attachment_4: FIRST SURVEY | 27 |
| 19. Attachment_5: INVITATION EMAIL SECOND SURVEY | 43 |
| 20. Attachment_6: SECOND SURVEY | 44 |
| 21. Attachment_7: INVITATION EMAIL CASE STUDY | 55 |
| 22. Attachment_8: PARTICIPANT INFORMATION CASE STUDY | 56 |
| 23. Attachment_9: INFORMED CONSENT FORM CASE STUDY | 58 |
| 24. Attachment_10: CASE STUDY INTERVIEW OUTLINE | 60 |
| 25. Attachment_11: INVITATION EMAIL SEMI-STRUCTURED INTERVIEW | 62 |
| 26. Attachment_12: PARTICIPANT INFORMATION SEMI-STRUCTURED INTERVIEW | 63 |
| 27. Attachment_13: INFORMED CONSENT FORM SEMI-STRUCTURED INTERVIEW | 65 |
| 28. Attachment_14: SEMI-STRUCTURED INTERVIEW OUTLINE | 67 |
| 29. Attachment_15: TRANSPARANCY STATEMENT DIABETS UK | 68 |

**LIST OF ABBREVIATIONS**

| CU Coventry University  GCP Good Clinical Practice  GDPR General Data Protection Regulation  ICF Informed Consent Form  ICH International Conference on Harmonisation of technical requirements for registration of pharmaceuticals for human use.  PI Participant Information  WMAHSN West Midlands Academic and Health  Science Network |  |
| --- | --- |
|  |  |

**PROJECT PROTOCOL**

Platform evaluation

# INTRODUCTION

1.1 Background

The prevalence of Type 2 Diabetes, and the number of people who are at risk for developing this condition, is rising every year. There are 4.7 million people suffering from this condition with 12.3 million people being at risk for developing Diabetes in the UK (Diabetes UK, 2020). Diabetes can have severe long-term health consequences but also increases health care costs (Jönsson, 2002). Diabetes UK (2020) offers an ecosystem with a variety of education tools through their website aimed at people with Type 1 Diabetes, Type 2 Diabetes, those who are at risk for developing Type 2 Diabetes and those who are caring for people with Diabetes. The platform developer developed an innovative personalised nutrition platform to support the delivery of healthy food recipes and meal planning for people with Diabetes.

- 1. Proposed project

This proposed project aims to assess the impact of the platform on important health outcomes and examine intervention outcomes across people with Type 1 or Type 2 Diabetes (or at risk) as referred by Diabetes UK through their website (<https://www.diabetes.org.uk/>). Doing so will allow the West Midland Academic Health and Science Network (WMAHSN) and Diabetes UK to take an informed decision as to whether a business case can be made to support the platform as part of the Diabetes UK ecosystem; and if so, provide supporting information for the business case for further roll-out based on the available evidence and insight gained.

1.3 Project population

Project participants consist of people with Type 1 or Type 2 Diabetes (or at risk) as referred by Diabetes UK through their website. Other stakeholders such as platform developer and Diabetes UK staff (i.e. dieticians) will be interviewed for implementation purposes and expert opinions.

1.4 Treatment / Intervention (if applicable)

The sample consists of people with Type 1 or Type 2 Diabetes or those who are at risk of developing Type 2 Diabetes. People will be compared on health and intervention outcomes before and after using the platform to see if there are differences in outcomes over time. This will allow to make within group comparisons using descriptive and inferential (where possible) statistics.

# 2. RATIONALE

- 1. Aims and hypothesis

This project aims to assess the impact of the platform on important health outcomes across those diagnosed with Diabetes Type 1 or Diabetes 2 (including those who are at risk for developing Type 2 Diabetes). Intervention outcomes will also be examined. There are no specific hypotheses formulated for the current project.

- 1. Justification

This proposed project aims to assess the impact of the platform on important health outcomes and examine intervention outcomes across people with Type 1 or Type 2 Diabetes (or at risk) as referred by Diabetes UK through their website (<https://www.diabetes.org.uk/>). Doing so will allow the West Midland Academic Health and Science Network (WMAHSN) and Diabetes UK to take an informed decision as to whether a business case can be made to support the platform as part of the Diabetes UK ecosystem; and if so, provide supporting information for the business case for further roll-out based on the available evidence and insight gained.

## Assessment and management of risk

## There are no ethical issues or significant risks that the project may lead to physical or emotional distress to participants or researchers or cause any reputational damage to the stakeholders involved. Given the research portfolio of Coventry University (CU) and that all project members are aware of the CU disclosure protocol, Code of Conduct and trained according to Good Clinical Practice (GCP) guidelines no management issues are expected. Also, the project includes a relatively small samples size compared to traditional trials which lowers the risk on management issues.

# 3 OBJECTIVES AND OUTCOME MEASURES/ENDPOINTS

# 3.1 Primary objective

The primary objective of the current project is to assess the impact of the platform on important health outcomes across those diagnosed with Diabetes Type 1 or Diabetes Type 2 (or for those who are at risk of Type 2 Diabetes). Intervention outcomes will also be examined.

The specific objectives of the current project are:

1. To assess whether incorporating the platform into the Diabetes UK ecosystem is associated with improved health and intervention outcomes in people with Diabetes Type 1, Diabetes Type 2 or people who are at risk for developing Diabetes Type 2 through analysing and comparing available data derived from surveys, Mixpanel for those engaging with the platform;
2. To examine if any possible observed impacts on health and intervention outcomes vary across participants differing in expectations regarding the platform, e.g. sex, diagnosis, age, ethnicity, digital literacy;
3. To assess platform usability data through Mixpanel and any potentially perceived benefits in terms of user experience of the platform, through case studies, semi-structured online interviews (including platform developer staff and Diabetes UK dieticians), and surveys; and assess potential weaknesses and disadvantages of the platform;
4. To assess if perceptions regarding Diabetes UK are changed after offering an innovative online nutrition tool for their users;
5. To describe and summarise results in intermediate and final reports including conclusions and recommendations on how to improve the platform and integration into Diabetes UK ecosystem as to the next steps and potential further roll out, as well as an executive summary.

3.2 Primary endpoint/outcome

The primary outcomes are categorized as follows: (1) Outcomes directly related to the health status and condition management of the participants (e.g. weight, BMI, HbA1c, blood pressure, waist, generic health status, smoking yes/no, confidence in diabetes management and meal planning, healthy eating) and (2) Outcomes directly related to platform intervention usage:

- Number / Duration of sessions
- Number of users / average sessions per user
- New users
- Monthly Active Users / Weekly Active Users
- Specific actions:
  - # Recipes Viewed, (any indication on favourite content)
  - # Recipe Added / Saved
  - # Meal Plans created
  - # Shopping Actions
    - Carts sent online
    - Meal plans sent to cart
    - In store shopping actions - print, email, sms, app, etc.
    - Number of items shopped / value
- Platform used - app vs web app
- GA audience metrics: Demographics, Geography,
- User Retention
- Top 20 recipes (potentially)
- The average or typical meal plan including health score (potentially)

Additionally, expectations beforehand and satisfaction afterwards will be assessed. Diabetes UK will collect data regarding their traffic on the website and the amount of referrals to the online platform.

# 4 PROJECT DESIGN

Health and intervention outcomes within the intervention group (before and after completing the platform) will be examined using a mixed-method research approach consisting of descriptive and inferential (where possible) statistics on surveys, Mixpanel data as well as thematic analyses on case studies and semi-structured online interviews.

# 5 PROJECT SETTING

The project will run fully online using Microsoft Teams application (including recording, storing and screen share options) where CU will invite participants for case studies and semi-structured interviews. This is seen as a good, safe and secure environment which will be comfortable and have ease of access for both participants and research staff.

**6 ELIGIBILITY CRITERIA**

The population consists of people with Type 1 or Type 2 Diabetes (or at risk) as referred by Diabetes UK through their website. Other stakeholders such as platform developer and Diabetes UK staff (i.e. dieticians) will be interviewed for implementation purposes and expert opinions.

**6.1 Inclusion criteria**

Project participants:

- Diagnosis Type 1
- Diagnosis Type 2 or at risk for Diabetes Type 2
- Relatives taking care of people with Type 1 or Type 2 Diabetes (or at risk)
- Good understanding of written and spoken English language

Platform development staff: Good understanding and know-how of platform and current project

Diabetes UK staff: Qualified and experienced dieticians working with people having Type 1 Diabetes, Type 2 Diabetes or those who are at risk for developing Type 2 Diabetes.

**6.2 Exclusion criteria**

There are no specific exclusion criteria formulated for the current project.

# 7 TRIAL PROCEDURES

Diabetes UK will refer potential participants through the Diabetes UK website to the platform where they can sign up, giving consent to platform terms and conditions as they sign in to use the Wisk platform whether or not they want to participate in the research (choosing not to participate in the research will not stop them from using the platform). They are also asked to give permission to share their email address and Mixpanel data to be passed on to the lead CU researchers allowing them to contact participants as part of the wider research project. It must be noted that when participants only want their email address to be shared and not their platform usage data they will still be able to participate into the research by filling in the two surveys as Mixpanel data would be labelled as missing at that point. The plaform developers will send email addresses of new users to lead CU researchers in a shared OneDrive folder with weekly updates. Mixpanel data, with email address as an identifying variable for each user, will be shared in a shared OneDrive folder on a weekly basis.

The lead CU researchers will send an invitation email to participants to fill in the first survey (please see Attachment 1) with enclosed Participant Information (PI; please see Attachment_2) and Informed Consent Form (ICF; please see Attachment_3) explaining the project aim, related activities and burden in terms of duration. The email will contain a link to the first survey (hosted by Qualtrics; please see Attachment_4) in which both PI and ICF are embedded. Two automated reminder emails will be send by Qualtrics in cases where participants do not fill in the first survey, one after a week of non-response, and one after two weeks of non-response. All data will be deleted in case there is non-response after the three emails. If participants do not want to be contacted or receive any reminder emails they can email they would like to UNSUBSCRIBE. Each participant will be provided with a unique survey link that recognises each participant by a unique response number only. The end of the first survey will present the option if the participant is (1) willing to participate into a prize draw (using their email address) and (2) willing to participate in follow-up research so selected participants can be approached for the case studies or semi-structured online interviews. After 8 weeks of using the platform, participants will receive an invitation email (please see Attachment_5) to fill in the second survey (please see Attachment_6).

Based on platform usage data (low vs. high engagement) and provided consent at the end of the first survey, participants will be selected for further research activities which consist of case study and semi-structured online interviews. Participants selected for the case study interview will receive an invitation email (please see Attachment_7) with PI (please see Attachment_8) and ICF enclosed. Participants who are willing to participate into the case study interview will sign the ICF (please see Attachment_9) and send it back to the CU lead researcher. Participants who prefer a phone interview can indicate this in an accompanying email. The case study interview outline is presented in Attachment_10.

Participants selected for the semi-structured interview will receive an invitation email (please see Attachment_11) with PI (please see Attachment_12) and ICF enclosed. Participants who are willing to participate into the semi-structured interview will sign the ICF (please see Attachment_13) and send it back to the CU lead researcher. Participants who prefer a phone interview can indicate this in an accompanying email. The semi-structured interview outline for service-users, platform developer staff and Diabetes UK staff is presented in Attachment_14.

The case studies and semi-structured interviews will be performed online through the Microsoft Teams application, supporting screen sharing as well as recording while using a Dictaphone for back-up recording. Data files will be automatically saved in Microsoft Streams and files recorded through the dictaphone will be stored immediately after the interview in a dedicated Onedrive folder so the recording can be deleted from dictaphone. Data will be destroyed after three years from project start date. Please see Figure 1 for the process of data sharing.

- 1. **Recruitment**

The sample will be recruited by Diabetes UK through their website (<https://www.diabetes.org.uk/>) supported by exposure through social media (e.g. <https://twitter.com/DiabetesUK>). The Diabetes UK website which will refer participants to the platform and accompanying opportunity to participate into the research project. The platform developer and CU will use their Twitter accounts to retweet messages from Diabetes UK. Recruitment will continue till 01/11/2020 so there is enough time to include the final participants into the analyses and therefore final report. Recruitment rates will be reviewed on a weekly basis*, allowing DiabetesUK to take action to promote the project further if required, or the recruitment time could be extended to allow for further new users and survey completions. Please see Attachment_15 for Diabetes UK transparency statement.

*To review recruitment rates, project partners will complete the spreadsheet below on a weekly basis:


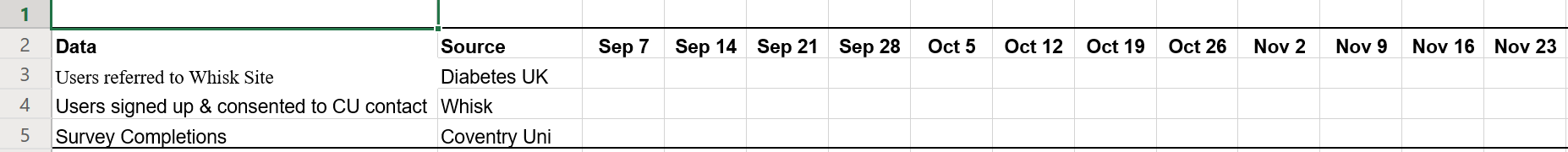


7.1.1 Patient identification

Diabetes UK will refer potential participants through the Diabetes UK website to the platform where they can sign up, giving permission to share Mix panel data (including their email address), to be passed on to the lead CU researchers allowing them to contact participants as part of the wider research project. The lead CU researchers will send an invitation email to participants with enclosed Participant Information and Informed Consent Form explaining the project aim, related activities and burden in terms of duration. Once participants provided informed consent electronically, survey questions will be shown for which a reminder will be send two times to non-responders (unless they reply on the email by unsubscribe) by an automated setting through Qualtrics. The first part of the survey will verify diagnosis (e.g., Type 1, Type 2, at risk or other) and sufficient understanding of verbal/written English language would be derived from completing the consent form.

- - 1. Screening

For the current project no laboratory or diagnostic testing is involved in the above-mentioned inclusion criteria. Therefore, no specific duration between screening and recruitment is expected. Based on the current inclusion/exclusion criteria, no participants will be expected to be subject to rescreening.

7.1.3 Payment

Case study and interview participants will be offered a £10 (interview) or £20 (case study) gift voucher as a thank you for giving up their time. Survey respondents will have the opportunity to enter a prize draw to win gift vouchers (£55 per survey) as a thank you for giving up their time. It is considered that these amounts will recognise and thank participants for giving up their time to contribute to the project, and are not amounts that would encourage undue inducement.


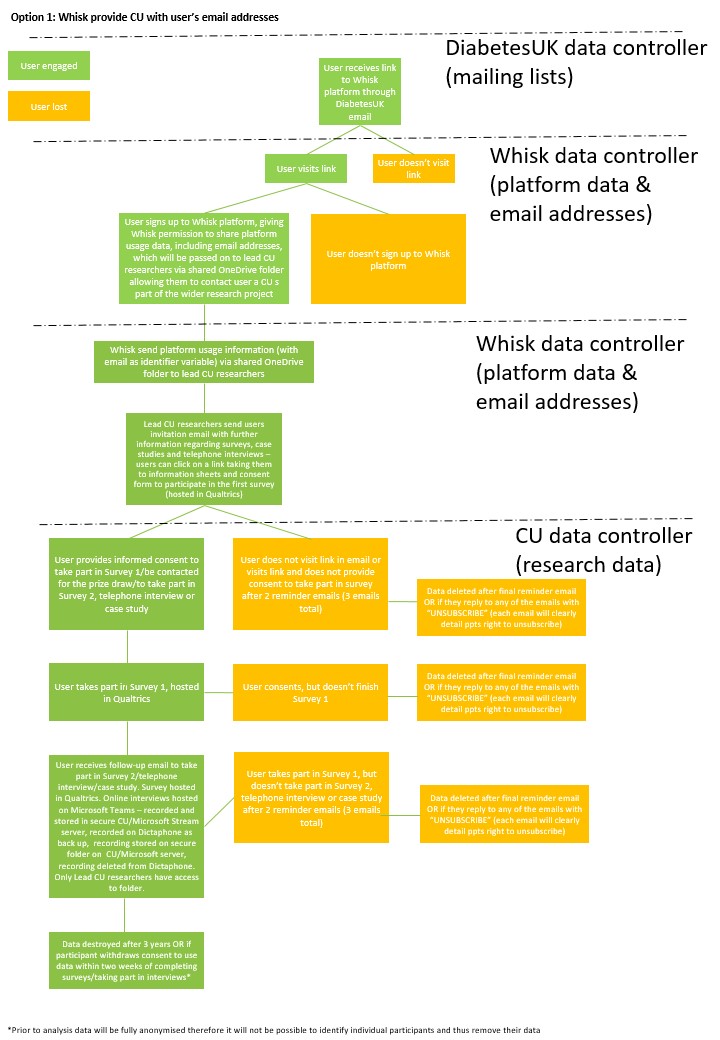


Figure 1. Flowchart data sharing

**7.2 Consent**

Once participants signed up for the platform, giving permission to share Mix Panel data and their email address, CU lead researchers will approach participants by email and shortly inform them about the project purposes, related activities and burden in terms of duration. The participant will receive the PI, ICF and Invitation Letter by email which they can sign electronically. These materials are in compliance with GCP guidelines, local regulatory requirements and legal requirements. Participants will have one week before they receive a reminder email, which is seen as an adequate amount of time given to decide if they want to participate or not. Participants will also be offered the possibility to ask additional questions regarding their participation in the current project.

It is important to stress that participants have a free choice to participate in the current project. The participant has the right to refuse participation and withdraw at any time without giving reasons why they do not want to participate. This will not influence his/her current or further participation in programmes delivered by Diabetes UK or platform developer. Participants will be informed that they may withdraw any time until the point that their data is anonymised for analysis, this will be two weeks after data collection took place. At this point the researchers will not be able to identify their data for withdrawal. CU lead researchers have to ensure that in case re-consent (or in case new information) is required this is done in a timely manner. All participants are required to sufficiently understand verbal/written English language. Participants will provide electronically signed informed consent as they are expected to have sufficient understanding to give full consent to participate in the current project independently.

**7.3 Randomisation scheme (if applicable)**

Not applicable.

**7.4 Baseline data**

For the current project, no data will be collected at baseline apart from data concerning inclusion/exclusion criteria, sociodemographic data, digital literacy, general health status, diabetes related health, confidence in meal planning, expectations regarding the platform, healthy eating. This will all be included in the first survey distributed through Qualtrics software.

**7.5 Trial assessments**

7.5.1 Descriptive and Inferential data analyses

Descriptive and inferential (where possible) analyses will be performed in IBM SPSS software (version 26) on health and intervention outcomes collected through survey and Mixpanel data. The survey will assess sociodemographic (e.g., diagnosis, age, gender, ethnicity, educational status, digital literacy) variables, health outcomes and will ask for expectations regarding the platform beforehand and satisfaction afterwards. Participants will be compared on health outcomes before and after the use of the platform to see if there are differences in health outcomes (e.g. weight, length, HbA1c, blood pressure, waist, generic health status, smoking yes/no) over time. Descriptive data of session frequency, session duration, pages visited, health score for recipes used on the platform as derived from Mixpanel will also be requested from platform developer to get insight into platform usage and support the selection of case studies. For Diabetes UK this will focus on traffic to site and referrals. Sub-group (or moderator analyses) according to expectations, age, ethnicity, diagnosis, digital literacy and sex will be conducted to examine possible differences in intervention and health outcomes. Data will be analysed using IBM SPSS Software, generating descriptive and inferential statistics with reported effect size, if data availability/quality allows.

7.5.2 Case study interviews

The project will include four case studies to gain a deeper understanding of the issues and challenges platform users encounter whilst using the platform and related education offered by Diabetes UK. The project team aims to include a variety of users for these case studies ranging from younger and older service users, British, Asian, Black, Mixed and other backgrounds but also in terms of intervention engagement (low versus high users). Where possible, the platform developer will provide data on hotspot analysis to guide the questions and key areas of the platform to look at. Preferred case studies will be identified with support from Diabetes UK. The case studies will give insight into how people use and engage with the platform (also during COVID-19 restrictions), and the factors contributing to platform engagement but also gives understanding to which platform elements could be improved. After obtaining ethical permission and informed consent, each selected case study will be interviewed individually and in-depth via online screen-sharing software (or telephone if preferred) for up to 1.5 hours. This will be video recorded through Microsoft Teams and audio-taped for back-up purposes. Participants will receive a gift voucher of £20 to thank them for giving up their time.

7.5.3 Online semi-structured interviews

Online semi-structured interviews (supported by individual telephone interviews where required) with relevant stakeholders: Online interviews will last between 30 minutes and 1-hour, utilising Microsoft Teams software which contains screen-sharing and recording options stored in Microsoft Stream. The screen sharing option is specifically useful to ensure understanding between researcher and participant when discussing specific aspects of the platform. Participants who are not comfortable with an online conversation will be offered a traditional telephone interview. In total, it will be anticipated to conduct 24 interviews with (1) Platform developer, Diabetes UK staff members; and (2) Platform users with a variety of sociodemographic characteristics as identified from survey results and usage patterns as identified from Mix panel. Results of these online interviews will be transcribed verbatim and thematically analysed (based on Thematic Analysis). Where possible, the platform will provide data on hotspot analysis to guide the questions and key areas of the platform to look at. Questions will also be developed to explore in more detail the findings of the survey. Key themes from the analysis will be presented in the interim and final reports. Interviews will be conducted until “theoretical saturation” is reached – the point where no new concepts emerge from interviews. Considering the variety of sociodemographic and type of diabetes to be included in the project, it is anticipated that this could be approximately 24 users, from which 4 are platform staff members and 4 are Diabetes UK dieticians. Participants will receive a gift voucher of £10 to thank them for giving up their time.

7.5.4 Surveys

Survey to all platform users invited to use the platform and familiar with Diabetes UK ecosystem: An online survey taking a maximum of 20 minutes to complete (hosted by Qualtrics) will be sent to all service users who were referred by Diabetes UK to use the platform and examine their user experience and possible difference in perceptions towards Diabetes UK. This survey will contain questions about expectations at study commencement, usefulness, satisfaction and ease of use, as well as exploration of reasons for how often they use the platform recipes (also during COVID-19 restrictions), as this may differ from tracked platform usage as captured by Mixpanel (e.g. if they printed off their recipes upon first login, therefore had no need to login to the platform again). The survey will include an adapted version of the System Usability Scale. Survey results will be exported to and coded using IBM SPSS software and described using descriptive statistics according to sociodemographic characteristics (e.g. ethnic group, age, digital literacy, educational level, diagnosis and sex) where possible. Participants will be entered into a gift voucher prize draw (to win a prize of £55) to thank them for giving up their time.

## **7.6 End of Project definition**

Data collection will end once the last participant completed the *after* survey. The project will end after the final report has been submitted to WMAHSN (estimated after a 12 months period).

# 8. STATISTICS AND DATA ANALYSIS

**8.1 Sample size calculation**

The current project consists of platform usage data (collected through Mixpanel), *before* and *after* surveys (anticipated n= 200-300), case studies (n=4) and online semi-structured interviews (n=24). Sample size is not derived statistically but based on the exploratory nature of the project and qualitative case study / interview set-up.

**8.2 Planned recruitment rate**

The planned recruitment rate for case study interviews is four participants and 24 participants for the online semi-structured interviews depending on the availability of platform developer staff (n=3) and Diabetes UK dieticians (n=3). All participants (anticipated n=200-300) will be approached to fill in a *before* and *after* survey. Overall, it is expected that more participants will be approached than mentioned above as nonresponse should be taken into account.

- 1. **Statistical analysis plan**

8.3.1 Summary of baseline data and flow of patients

These baseline data consist of height, weight, waist, BMI, blood pressure, Hba1c, sex, ethnicity, religion, employment status, sexual orientation, accommodation status, disability status, smoking status and general health status and expectations regarding the platform.

8.3.2 Primary outcome analysis

Participants will be analysed on health outcomes before and after they used the platform to see if there is an improvement within this group over time. These data will be analysed using IBM SPSS software (version 26) generating descriptive and inferential statistics with reported effect size (if data availability/quality allows). Descriptive data of platform usage (e.g. session frequency, session duration, pages visited, health score for recipes used) will also be provided by the platform developer through Mix panel. These data will be exported into IBM SPSS software (version 26) and will be used to select participants with different engagement levels (low vs. high) for participation in case studies and semi-structured interviews. Where possible descriptive data of platform usage will be related to health outcomes. Results of the case study interviews and semi-structured interviews will be transcribed verbatim and thematically analysed (based on Grounded Theory) resulting in overall key themes. Survey results will be exported to and coded using IBM SPSS software (version 26) and described using descriptive statistics (e.g. frequencies, percentages, medians and means) according to sociodemographic characteristics (e.g. ethnic group, age, digital literacy, educational level, diagnosis and sex).

**8.4** **Subgroup analyses**

Sub-group (or moderator analyses) according to expectations, age, ethnicity, digital literacy and sex will be conducted to examine possible differences in intervention and health outcomes. Data will be analysed using IBM SPSS Software, generating descriptive and inferential statistics with reported effect size, if data availability/quality allows.

**8.5 Participant population**

Any participant who participated in the project will be subjected to statistical or thematic analyses, except for participants who withdraw from the project before data gets anonymized. This date will be explicitly mentioned in the Participant Information and will be two weeks after data collection took place. Moreover, participants who did not opt-in for their platform usage data to be shared with CU lead researchers upon referral to the platform through Diabetes UK will not be subject to statistical analyses and labelled as missing data.

**8.6 Procedure(s) to account for missing or spurious data**

The semi-structured interviews and case studies will be video and audio recorded. Participants will be informed that they may withdraw any time up until the point that their data is anonymised for analysis. At this point the researchers will not be able to identify their data for withdrawal. This date will be two weeks after the semi-structured interview / case study has taken place and will be mentioned in the Participant Information.

# 9 DATA MANAGEMENT

# 9.1 Data collection tools and source document identification

Diabetes UK will refer potential participants through the Diabetes UK website to the platform where they can sign up allowing CU researchers them to contact participants as part of the wider research project. The case studies and semi-structured interviews will be performed online through the Microsoft Teams application, supporting screen sharing as well as recording while using a Dictaphone for back-up recording. Data files will be automatically saved in Microsoft Streams and files recorded through the dictaphone will be stored immediately after the interview in a dedicated OneDrive folder so the recording can be deleted from dictaphone. Survey data (incl. informed consent) will be collected online through Qualtrics, hosted by CU and will be retained in shared OneDrive which only the project team has access to. CU is responsible to keep records of all participants, all electronically signed ICFs in a shared OneDrive folder that is only accessible by the project team at CU. Disposal date of these data is 3 years from project start.

## **9.2 Data handling and record keeping**

As required by the GCP guidelines CU will ensure that a validated system is used, an audit trial of data changes is maintained ensuring that there is no deletion of entered data, a security system is maintained to protect against unauthorized access, a list of the individuals authorized to make data changes is maintained, adequate backup of the data is maintained and archiving of any source data (i.e. hard copy and electronic) is adequately performed. As such, it will always be possible to compare the original data with the processed data. An unambiguous participant identification code will be used to allow identification of all the data reported for each participant.

## **9.3 Access to Data**

Direct access will be granted to authorised representatives from the Funder, host institution and the regulatory authorities to permit project-related monitoring, audits and inspections - in line with participant consent.

9.4 Archiving

Disposal date of personal data is six months from project start and three years from project start date for remaining data. As there will not be no hard-copy data no archiving is needed.

**10. TRIAL OVERSIGHT**

**10.1 Role and responsibilities of the Sponsor**

WMAHSN is the sponsor for this project and will undertake the responsibilities of sponsor as defined by the Research Governance Framework and ICH GCP. An authorised representative of the Sponsor has approved the final version of this protocol with respect to the project design, conduct, data analysis and interpretation.

**10.2 Role and responsibilities of the Funder**

Funding for this trial is provided by WMAHSN. The design and management of this project are entirely independent of the funder and are performed by CU lead researchers.

**10.3 TRIAL MANAGEMENT ARRANGEMENTS**

The project lead will have responsibility for overseeing day to day coordination of the project and reporting regularly to the project lead. The project lead responsibilities include, but are not limited to:

- Coordinating protocol development, participant and project management documents
- Correspondence with project funder and tracking of progress against agreed key performance indicators
- Ensuring necessary approvals are in place before project start;
- Providing data management support; including data input, maintenance of the project database and raising of queries
- Producing in-between progress reports and coordinating internal/external meetings and minutes;
- Ensuring data security and quality and ensuring data protection laws are adhered to;
- Ensuring complete records are in place for audit and monitoring purposes;
- Ensuring the project is conducted in accordance with the ICH GCP.

### 11. MONITORING, AUDIT & INSPECTION

The project will be monitored by the Research Governance team at CU, to ensure that the project is being conducted as per protocol, adhering to Research Governance and GCP. The approach to, and extent of, monitoring has been specified in the Information Governance Unit determined by the risk assessment undertaken prior to the start of the project.

# 12. ETHICAL AND REGULATORY CONSIDERATIONS

**12.1 Ethical approval and research governance**

The project will be conducted in compliance with the principles of the ICH GCP guidelines and in accordance with all applicable regulatory guidance, including, but not limited to, the UK policy framework for health and social care research. Ethical approval for this project will be sought from the CU Research Ethics Committee. No project activities will commence until favourable ethical opinion has been obtained. The final report at the conclusion of the project will be submitted to the approving CU Research Ethics Committee.

**12.2 Peer review**

The current project has not undergone independent peer review but has been reviewed by CU Research Ethics Committee as well as partners from platform developer, Diabetes UK and WMAHSN.

**12.3 Public and Patient Involvement**

Within the context of the current project no public and patient involvement activities will be performed to decide upon project aim, design etc.

**12.4 Data protection and patient confidentiality**

The project will comply with the GDPR and regular checks and monitoring will be undertaken by the project lead to ensure compliance. Participants have been and will be assigned a unique identifier upon enrolment in to the project to allow link-anonymisation of participant-identifiable data. Access to patient identifiable data will be restricted to members of the project team who require it for the performance of their role. A GDPR acknowledged and approved transcribing service (called Just Delegate) will be used to type out case study interviews and online semi-structured interviews in verbatim. Electronic data (i.e. survey results) will be stored on shared OneDrive folder and hard copies of project documents will be stored in locked filing cabinets in secure entry-card protected sites at CU.

**13. DISSEMINATION POLICY**

Data resulting from the current project are owned by WMAHSN but the funder grants CU licence to use materials for academic research, teaching and education purposes. An intermediate and final project report will be written by the involved project team members of CU. Participants will receive a short anonymised summary report of the main findings. This will be lay people friendly and contains non-commercially sensitive findings. In case an anonymized case study will be published, this will need to be agreed upon by all project partners.

**14. REFERENCES**

Brooke, J. (1996). SUS-A quick and dirty usability scale. *Usability evaluation in industry*, *189*(194), 4-7.

Diabetes UK (2018, February). *Number of people living with diabetes doubles in twenty years.* Retrieved from <https://www.diabetes.org.uk/about_us/news/diabetes-prevalence-statistics>

Jönsson, B. (2002). Revealing the cost of Type II diabetes in Europe. *Diabetologia*, *45*(1), S5-S12.

**15. Attachment_1: Invitation Email to participate in research project “platform evaluation”**

<Insert date>

Dear <Insert participant name>,

**Re: Invitation to participate in research project “platform evaluation”**

This invitation email has been sent to you after you signed up for the platform <Insert Link> and we, Coventry University lead project researchers, received your email address to contact you and see if you are interested in participating in our research project.

You will be asked to fill in two surveys, one survey before you start using the platform and one survey after you used the platform for a period of 8 weeks. Each survey will take approximately 20 minutes to fill in.

Before you fill in the first survey you will be presented with Participant Information, which provides more details about the project. Since you are eligible to participate in the project, I would be grateful if you could read the PI and discuss this with your family and friends if you wish. If you agree to participate in this project, you can consent by clicking YES against each of the statements presented on the Informed Consent Form (ICF), which will follow the information, and then signing and dating the form as a participant.

You can access the first survey here: <Insert Link>

You are under no obligation to participate. There will be no consequences of not taking part. If you have any further questions, please do not hesitate to contact the project lead on [kim.bul@coventry.ac.uk](mailto:kim.bul@coventry.ac.uk)

I would like to thank you for your time and consideration.

Yours Sincerely,

Dr. Kim Bul

Nikki Holliday

Rachael Molitor

Coventry University

**16. Attachment_2: Participant Information (presented in Qualtrics)**

**Project title**: **Platform evaluation**

**Project lead: Dr. Kim Bul**

**Local project team: Nikki Holliday, Rachael Molitor**

Dear Participant,

You are being invited to take part in research to explore how the platform can support people with Diabetes or people who take care of people with this condition in their meal planning. Dr. Kim Bul, Research Fellow at Coventry University, is leading this research. Before you decide to take part, it is important you understand why the research is being conducted and what it will involve. Please take time to read the following information carefully.

**What is the purpose of the study?**

The aim of this study is to assess the impact of the platform, an online nutrition tool for people with Diabetes Type 1, Diabetes Type 2, who are at risk of developing Diabetes Type 2 or for people who are caring for a person with this condition. We are looking to find out how people use the platform, and if it helps them manage their diabetes and meal planning.

**Why have I been chosen to take part?**

You have been selected to take part in two surveys because you have been using the platform and have agreed to be approached by us for the research project.

**What are the benefits of taking part?**

This project provides you and other people who use the platform the opportunity to share your experiences of using it. There will not be a direct benefit for you as an individual, but your answers will contribute to further development and improvement of the platform to support people with Diabetes Type 1, Diabetes Type 2, those who are at risk or taking care of someone with this condition.

**Are there any risk with taking part?**

This study has been reviewed and approved through Coventry University’s formal research ethics procedure (Insert ref number and date). There are no significant risks associated with participation. We ask that you complete all of the questions to aid our research or indicate “prefer not to say” where applicable.

**Do I have to take part?**

Your participation in both surveys is entirely voluntary, and you can opt out at any stage by closing and exiting the browser.

**What will happen if I decide to take part?**

You will be asked to fill in two surveys, one survey before you start using the platform (which will appear after you have agreed to take part) and one survey after you used the platform for a period of 8 weeks (we will email a link to you). Each survey will take approximately 20 minutes to fill in. You may have also consented to the platform securely sending information about how often you use the platform. We will use this to understand how people use the platform. Results will be used to further develop and improve the platform in the future. No individual participants will be identified when the results are presented. Every participant will receive a final summary report of the results.

**Will my taking part in the research project be kept confidential?**

Your survey answers will be treated confidentially and the information you provide will be kept anonymous in any research outputs/publications. Your data will be processed in accordance with the General Data Protection Regulation 2016 (GDPR) and the Data Protection Act 2018. Your data will be held securely and will only be viewed by the researcher/research team. Personally identifiable data will be deleted 6 months after project start and anonymized data will be deleted three years after project start.

**What will happen if I don't want to carry on with the project?**

You are free to withdraw your survey responses from the project data set at any time until the data are fully anonymised in our records on [ENTER DATE]. You should note that your data may be used in the production of formal research outputs (e.g. journal articles, conference papers, theses and reports) prior to this date and so you are advised to contact the university at the earliest opportunity should you wish to withdraw from the study. To withdraw, please contact the lead researcher (contact details are provided below). Please also contact the Faculty Research Support Office (email hls.rso@coventry.ac.uk; telephone +44(0)2477658461) so that your request can be dealt with promptly in the event of the lead researcher’s absence. You do not need to give a reason. A decision to withdraw, or not to take part, will not affect you in any way.

**Data protection rights**

Coventry University is a Data Controller for the information you provide. If consented on the platform landing page, the platform will securely send your usage data to Coventry University so we can see how often you used the platform. You have the right to access information held about you. Your right of access can be exercised in accordance with the General Data Protection Regulation and the Data Protection Act 2018. You also have other rights including rights of correction, erasure, objection, and data portability. For more details, including the right to lodge a complaint with the Information Commissioner’s Office, please visit [www.ico.org.uk](http://www.ico.org.uk) Questions, comments and requests about your personal data can also be sent to the University Data Protection Officer - [enquiry.ipu@coventry.ac.uk](mailto:enquiry.ipu@coventry.ac.uk)

**Will I be reimbursed for taking part?**

All participants will have the opportunity to enter a prize draw for completing two surveys and will receive a £55 shopping voucher when winning the first prize.

**Who is organising and funding the study?**

The West Midlands Academic Health Science Network (hosted by Birmingham NHS Foundation Trust) is funding the current research project.

**Further information and making a complaint**

For further information, or if you have any queries, please contact the lead researcher Dr. Kim Bul, [kim.bul@coventry.ac.uk](mailto:kim.bul@coventry.ac.uk). If you have any concerns that cannot be resolved through the lead researcher, please contact my line manager Professor John Allen ([ad5325@coventry.ac.uk](mailto:ad5325@coventry.ac.uk)). Thank you for taking the time to participate in this survey. Your help is very much appreciated.

**17. Attachment_3: Informed Consent Form (presented in Qualtrics)**

Having now read the previous information page, please do not hesitate to ask questions (kim.bul@coventry.ac.uk) if anything is unclear or if you would like more information about any aspect of this research. It is important that you feel able to take the necessary time to decide whether or not you wish to take part.

If you are happy to participate, please confirm your consent by clicking YES against each of the below statements and then signing the form. You will only be able to continue to the survey by selecting and agreeing to each of the statements below:

| **No.** | **Statement** | **NO** | **YES** |
| --- | --- | --- | --- |
| 1 | I confirm that I have read and understood the Participation Information for the study. I had the opportunity to consider the information, ask questions and have had these answered to my satisfaction. |  |  |
| 2 | I understand that my participation is voluntary and that I am free to withdraw before my information is anonymised on (Insert Date) without giving any reason, without my (medical) care or rights being affected. |  |  |
| 3 | I understand that all information I provide will be held securely and treated confidentially. |  |  |
| 4 | Should I choose to withdraw consent, I agree that information obtained from me in this research project up to that point may still be used (if not already anonymized). |  |  |
| 5 | I am happy for the information I provide to be used (anonymously) in academic papers and other research outputs. |  |  |
| 6 | I agree to take part in this project. |  |  |

Please sign to say you agree to the above and wish to continue.

*When completed: 1 electronic copy for the participant and keep the original in the project OneDrive folder.*

**18. Attachment_4: First Survey**

*Please complete as much information as possible about yourself (or the one you are taking care of) in the survey below.* *All information will be treated in confidence and nothing will be linked to you.*

**Sociodemographic background**

1. What is your current age?

_________________________years old

1. What is your gender?

□ Female

□ Male

□ Other, please describe

□ Prefer not to say

1. What is your ethnicity?

□ White

□ English/Welsh/Scottish/Northern Irish/British

□ Irish

□ Gypsy or Irish Traveller

□ Any other white background, please describe ________________

□ Mixed/Multiple ethnic groups

□ White and Black Caribbean

□ White and Black African

□ White and Asian

□ Any other Mixed/Multiple ethnic background, please describe ___________

□ Asian/Asian British

□ Indian

□ Pakistani

□ Bangladeshi

□ Chinese

□ Any other Asian background, please describe ____________

□ Black/African Caribbean/Black British

□ African

□ Caribbean

□ Any other Black/African/Caribbean background, please describe

□ Other ethnic group

□ Arab

□ Any other ethnic group, please describe

□ Any other ethnic group, please describe _________________________

□ Prefer not to say

1. What is your religion?

□ No religion

□ Christian

□ Buddhist

□ Hindu

□ Jewish

□ Muslim

□ Sikh

□ Any other religion (please state_______________________________________)

□ Prefer not to say

1. What is your relationship to Diabetes?

□ Type 1 diabetes

□ Type 2 diabetes

□ Gestational diabetes

□ I have a relative with Type 1 diabetes

□ I have a relative with Type 2 diabetes

□ Other type of diabetes, please describe _________________________

□ Prefer not to say

1. What is your current employment status? (if other please specify)

□ Full-time

□ Part-time

□ Student

□ Unemployed

□ Unable to work

□ Other, please specify_________________________

□ Prefer not to say

1. What is your education level?

□ High/Secondary school

□ College

□ University - Undergraduate

□ University - Postgraduate

□ Other, please specify_________________________

□ Prefer not to say

1. What is your marital status?

□ Never married or never registered in a civil partnership

□ Married or in a civil partnership

□ Divorced

□ Separated

□ Widowed

□ Cohabiting

□ Single

□ Prefer not to say

1. How many people are currently in your household?

□ 1

□ 2

□ 3

□ 4

□ 4+

□ Prefer not to say

1. Where do you live?

□ England

□ Northern Ireland

□ Scotland

□ Wales

□ Other (please specify)

□ Prefer not to say

1. Do you have any long-standing illness, disability or infirmity? By long-standing I mean anything that has troubled you over a period of time or that is likely to affect you over a period of time?

□ No

□ Yes

□ Prefer not to say

If yes, does this illness/illnesses or disability/disabilities limit your daily activities in any way?

□ No

□ Yes

□ Prefer not to say

**Device Usage**

1. On what device do you use the platform?

□ On an Android smartphone/tablet

□ On an Apple iPhone/iPad

□ On a computer with internet

2. Do you have mobile internet and/or wifi on your computer/smartphone/tablet?

□ Yes, I have mobile internet

□ Yes, I have wifi connection

□ Yes, I have mobile internet and wifi connection

□ No, I have none of them

3. For what purpose do you use your computer/smartphone/tablet the most?

□ Social media (like facebook, twitter, instagram)

□ Call/text/whatsapp

□ Listen to music (like Spotify, Youtube)

□ Search for information (like Google)

□ Other, please specify_________________________

4. Do you use your computer/smartphone/tablet to monitor and/or improve physical activity?

□ No

□ Yes, please specify_________________________

5. Do you use your computer/smartphone/tablet to monitor and/or improve your nutrition?

□ No

□ Yes, please specify_________________________

6. Do you use your computer/smartphone/tablet to monitor and/or improve your blood glucose level?

□ No

□ Yes, please specify_________________________

**Computer Proficiency Questionnaire – 12 (CPQ-12)**

This questionnaire asks about your ability to perform a number of tasks with a computer. Please answer each question by placing an X in the box that is most appropriate. If you have not tried to perform a task or do not know what it is, please mark “NEVER TRIED”, regardless of whether or not you think you may be able to perform the task.

**Computer/Smartphone/Tablet Basics**

| I can | Never tried_1_ | Not at all_2_ | Not very easily_3_ | Somewhat easily_4_ | Very easily_5_ |
| --- | --- | --- | --- | --- | --- |
| Use a computer/smartphone/tablet keyboard to type |  |  |  |  |  |
| Use a mouse or navigate on a smartphone/tablet |  |  |  |  |  |

**Printer**

| I can | Never tried_1_ | Not at all_2_ | Not very easily_3_ | Somewhat easily_4_ | Very easily_5_ |
| --- | --- | --- | --- | --- | --- |
| Load ink into the printer |  |  |  |  |  |
| Fix the printer when paper jams |  |  |  |  |  |

**Communication**

| I can | Never tried_1_ | Not at all_2_ | Not very easily_3_ | Somewhat easily_4_ | Very easily_5_ |
| --- | --- | --- | --- | --- | --- |
| Open emails |  |  |  |  |  |
| Send emails |  |  |  |  |  |

**Internet**

| I can | Never tried_1_ | Not at all_2_ | Not very easily_3_ | Somewhat easily_4_ | Very easily_5_ |
| --- | --- | --- | --- | --- | --- |
| Find information about local community resources on the Internet |  |  |  |  |  |
| Find information about my hobbies and interests on the Internet |  |  |  |  |  |

**Calendar**

| I can | Never tried_1_ | Not at all_2_ | Not very easily_3_ | Somewhat easily_4_ | Very easily_5_ |
| --- | --- | --- | --- | --- | --- |
| Use a computer/smartphone/tablet to enter events and appointments into a calendar |  |  |  |  |  |
| Check the date and time of upcoming and prior appointments |  |  |  |  |  |

**Entertainment**

| I can | Never tried_1_ | Not at all_2_ | Not very easily_3_ | Somewhat easily_4_ | Very easily_5_ |
| --- | --- | --- | --- | --- | --- |
| Use a computer/smartphone/tablet to watch movies and videos |  |  |  |  |  |
| Use a computer/smartphone/tablet to listen to music |  |  |  |  |  |

**General Health Status**

We would like to know how good or bad your health is TODAY. The scale is numbered from 0 to 100. 100 means the best health you can imagine. 0 means the worst health you can imagine. Mark an X on the scale to indicate how your health is TODAY. Now, please write the number you marked on the scale in the box below.

Your health today =


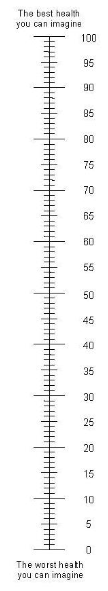


**Diabetes Related Health Status**

1. We would like to know your height. Would you like to answer in imperial (feet, inches) or metric (metres, centimetres) units? Please specify date DD/MM/YYYY.

□ Imperial (feet, inches)

□ Metric (metres, centimetres)

[Based on answer skip to drop down menus to select height in preferred units]

1. **How was this height measured:** (if other please specify)

□ Myself

□ Doctor

□ Other, please specify_________________________

□ Don’t know

□ Prefer not to say

1. We would like to know your weight. Would you like to answer in imperial (stones, pounds) or metric (kilograms) units? Please specify date DD/MM/YYYY.

□ Imperial (stones and pounds)

□ Metric (kilograms)

[Based on answer skip to drop down menus to select weight in preferred units]

1. **How was this weight measured:** (if other please specify)

□ Myself

□ Doctor

□ Other, please specify_________________________

□ Don’t know

□ Prefer not to say

1. We would like to know your waist size. Would you prefer to answer in imperial (inches) or metric units (centimetres)? Please specify date DD/MM/YYYY.

□ Imperial (inches)

□ Metric (centimetres)

[Based on answer skip to drop down menus to select waist size in preferred units]


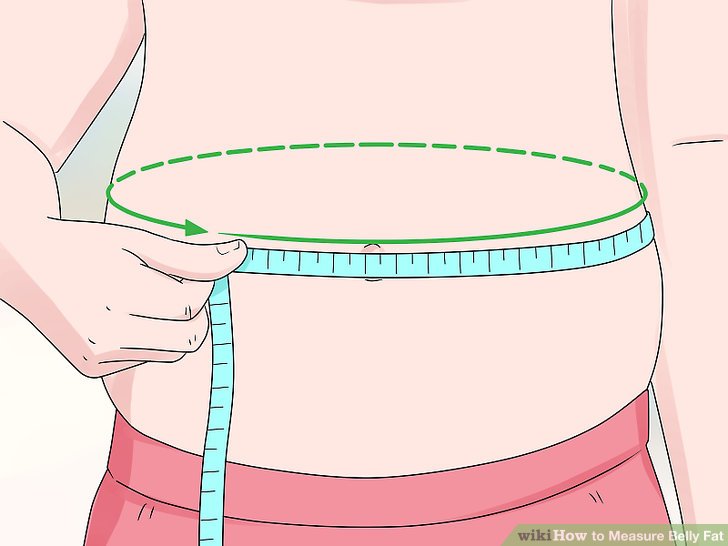
Above the belly button, just below the rib cage. Bend to one side to find natural crease of your waist. Measure across at this point.

1. **How was this waist size measured:** (if other please specify)

□ Myself

□ Doctor

□ Other, please specify_________________________

□ Don’t know

□ Prefer not to say

1. What is your most recent blood glucose (sugar) level? (HbA1c mmol/mol)

□ Don’t know

□ Prefer not to say

1. What is the date of your most recent blood glucose level measurement?

DD/MM/YYYY

□ Don’t know

□ Prefer not to say

1. What is your most recent blood pressure level?

Systolic (top number) mmHg [drop down menu – 70, 80, 90, 100, 110, 120, 130, 140, 150, 160, 170, 180, 190]

Diastolic (bottom number) mmHg [drop down menu – 40, 50, 60, 70, 80, 90, 100]

□ Don’t know

□ Prefer not to say

1. What was the date of your most recent blood pressure level?

DD/MM/YY

□ Don’t know

□ Prefer not to say

1. What was your most recent HDL cholesterol level (mmol/L)?

[drop down menu]

□ Don’t know

□ Prefer not to say

1. What was your most recent TOTAL cholesterol level?

______________ mmol/L [drop down menu]

□ Don’t know

□ Prefer not to say

1. What is the date of your most recent cholesterol measurement?

DD/MM/YYYY

□ Don’t know

□ Prefer not to say

1. Do you smoke?

□ Yes

□ No

**Confidence regarding diabetes management and meal planning**

1. How confident do you currently feel in managing your diabetes?

| 1 – Very unconfident | 2 | 3 | 4 | 5 – Neither confident nor unconfident | 6 | 7 | 8 | 9 | 10 – Very confident |
| --- | --- | --- | --- | --- | --- | --- | --- | --- | --- |
|  |  |  |  |  |  |  |  |  |  |

1. How confident do you currently feel in making healthy food choices?

| 1 – Very unconfident | 2 | 3 | 4 | 5 – Neither confident nor unconfident | 6 | 7 | 8 | 9 | 10 – Very confident |
| --- | --- | --- | --- | --- | --- | --- | --- | --- | --- |
|  |  |  |  |  |  |  |  |  |  |

1. How confident do you currently feel in planning meals efficiently?

| 1 – Very unconfident | 2 | 3 | 4 | 5 – Neither confident nor unconfident | 6 | 7 | 8 | 9 | 10 – Very confident |
| --- | --- | --- | --- | --- | --- | --- | --- | --- | --- |
|  |  |  |  |  |  |  |  |  |  |

**Healthy eating**

1. Do you eat at least 2 portions (200g) of fruit everyday?

□ Always

□ Often

□ Sometimes

□ Never

1. Do you eat at least 2 portions (200g) of vegetables everyday?

□ Always

□ Often

□ Sometimes

□ Never

1. Your diet:

□ Is different everyday

□ Is different only sometimes during a week

□ Is different only during weekend days

□ Is very monotonous

1. Do you drink at least 1 – 1.5 litre mineral water every day?

□ Always

□ Often

□ Sometimes

□ Never

1. Your snacks are based mainly on:

□ Fruit/fruit juice and milk shakes/yoghurt

□ Biscuits/crackers/bread/stick bread

□ Fried patatos/popcorn/krapfen/peanuts/soft drinks

□ Sweets/chocolate/ice cream/cakes

1. Do you eat breakfast?

□ Always

□ Often

□ Sometimes

□ Never

1. How many times do you eat sweets and cakes in 1 week?

□ 1-2

□ 3-4

□ 1 time a day

□ more than 1 time daily

□ 1 time in 10-15 days

□ never

1. How many times do you eat fish in 1 week?

□ 1-2

□ 3-4

□ 1 time a day

□ more than 1 time daily

□ 1 time in 10-15 days

□ never

**Expectations**

1. I think that the platform will help me to manage my diabetes

| 1 – Very Strongly disagree | 2 | 3 | 4 | 5 – Neither agree nor disagree | 6 | 7 | 8 | 9 | 10 – Very strongly agree |
| --- | --- | --- | --- | --- | --- | --- | --- | --- | --- |
|  |  |  |  |  |  |  |  |  |  |

Please explain your rating _________________________

1. The platform will help me to make healthy food choices

| 1 – Very Strongly disagree | 2 | 3 | 4 | 5 – Neither agree nor disagree | 6 | 7 | 8 | 9 | 10 – Very strongly agree |
| --- | --- | --- | --- | --- | --- | --- | --- | --- | --- |
|  |  |  |  |  |  |  |  |  |  |

Please explain your rating _________________________

1. The platform will help me to plan my meals more efficiently

| 1 – Very Strongly disagree | 2 | 3 | 4 | 5 – Neither agree nor disagree | 6 | 7 | 8 | 9 | 10 – Very strongly agree |
| --- | --- | --- | --- | --- | --- | --- | --- | --- | --- |
|  |  |  |  |  |  |  |  |  |  |

Please explain your rating _________________________

1. Using the platform will improve my food shopping experience

| 1 – Very Strongly disagree | 2 | 3 | 4 | 5 – Neither agree nor disagree | 6 | 7 | 8 | 9 | 10 – Very strongly agree |
| --- | --- | --- | --- | --- | --- | --- | --- | --- | --- |
|  |  |  |  |  |  |  |  |  |  |

Please explain your rating _________________________

**Thank you for filling in the first survey.**

You will be send a link to the second survey (hosted by Qualtrics) in 8 weeks.

If you are happy to participate into the prize draw and being possibly contacted for future interviews through email, please confirm your consent by clicking YES against each of the below statements:

| **No.** | **Statement** | **NO** | **YES** |
| --- | --- | --- | --- |
| 1 | I agree that I am happy to enter into the prize draw for the first and second survey participation based on my email address |  |  |
| 2 | I agree to be contacted by Coventry University for follow-up research activities (e.g. case study, semi-structured interview, or a phone interview alternatively) for which I may be suitable. I understand that agreeing to be contacted does not oblige me to participate in any activities. |  |  |

If you agreed to participate into the prize draw, you will find out through email if you won the

first prize for participating in the first survey.

If you require further information please contact project lead Dr. Kim Bul on [kim.bul@coventry.ac.uk](mailto:kim.bul@coventry.ac.uk).

**19. Attachment_5: Invitation Email to participate in second survey “Platform evaluation”**

<Insert date>

Dear <Insert participant name>,

**Re: Invitation to participate in second survey “Platform evaluation”**

You have been using the platform for about 8 weeks and we would like to know what your experiences are by filling in a second survey.

You can access the second survey here: <Insert Link>

This will take approximately 20 minutes to fill in.

You are under no obligation to participate. There will be no consequences of not taking part. If you have any further questions, please do not hesitate to contact the project lead on [kim.bul@coventry.ac.uk](mailto:kim.bul@coventry.ac.uk)

I would like to thank you for your time and consideration.

Yours Sincerely,

Dr. Kim Bul

Nikki Holliday

Rachael Molitor

Coventry University

**20. Attachment_6: Second Survey**

*Please complete as much information as possible about yourself (or the one you are taking care of) in the survey below.* *All information will be treated in confidence and nothing will be linked to you.*

**General Health Status**

We would like to know how good or bad your health is TODAY. The scale is numbered from 0 to 100. 100 means the best health you can imagine. 0 means the worst health you can imagine. Mark an X on the scale to indicate how your health is TODAY. Now, please write the number you marked on the scale in the box below.

Your health today =


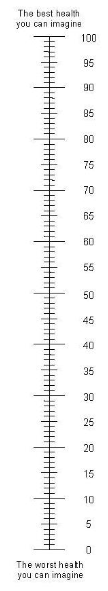


**Diabetes Related Health Status**

1. We would like to know your height. Would you like to answer in imperial (feet, inches) or metric (metres, centimetres) units? Please specify date DD/MM/YYYY.

□ Imperial (feet, inches)

□ Metric (metres, centimetres)

[Based on answer skip to drop down menus to select height in preferred units]

1. **How was this height measured:** (if other please specify)

□ Myself

□ Doctor

□ Other, please specify_________________________

□ Don’t know

□ Prefer not to say

1. We would like to know your weight. Would you like to answer in imperial (stones, pounds) or metric (kilograms) units? Please specify date DD/MM/YYYY.

□ Imperial (stones and pounds)

□ Metric (kilograms)

[Based on answer skip to drop down menus to select weight in preferred units]

1. **How was this weight measured:** (if other please specify)

□ Myself

□ Doctor

□ Other, please specify_________________________

□ Don’t know

□ Prefer not to say

1. We would like to know your waist size. Would you prefer to answer in imperial (inches) or metric units (centimetres)? Please specify date DD/MM/YYYY.

□ Imperial (inches)

□ Metric (centimetres)

[Based on answer skip to drop down menus to select waist size in preferred units]


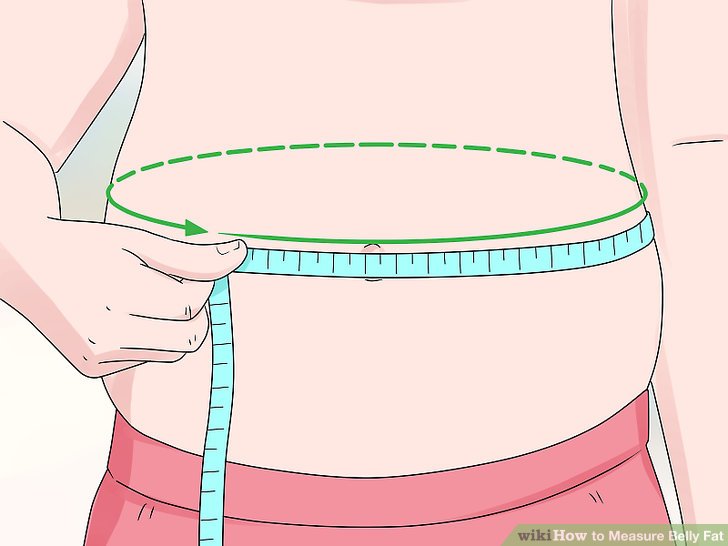
Above the belly button, just below the rib cage. Bend to one side to find natural crease of your waist. Measure across at this point.

1. **How was this waist size measured:** (if other please specify)

□ Myself

□ Doctor

□ Other, please specify_________________________

□ Don’t know

□ Prefer not to say

1. What is your most recent blood glucose (sugar) level? (HbA1c mmol/mol)

□ Don’t know

□ Prefer not to say

1. What is the date of your most recent blood glucose level measurement?

DD/MM/YYYY

□ Don’t know

□ Prefer not to say

1. What is your most recent blood pressure level?

Systolic (top number) mmHg [drop down menu – 70, 80, 90, 100, 110, 120, 130, 140, 150, 160, 170, 180, 190]

Diastolic (bottom number) mmHg [drop down menu – 40, 50, 60, 70, 80, 90, 100]

□ Don’t know

□ Prefer not to say

1. What was the date of your most recent blood pressure level?

DD/MM/YY

□ Don’t know

□ Prefer not to say

1. What was your most recent HDL cholesterol level (mmol/L)?

[drop down menu]

□ Don’t know

□ Prefer not to say

1. What was your most recent TOTAL cholesterol level?

______________ mmol/L [drop down menu]

□ Don’t know

□ Prefer not to say

1. What is the date of your most recent cholesterol measurement?

DD/MM/YYYY

□ Don’t know

□ Prefer not to say

1. Do you smoke?

□ Yes

□ No

**Confidence regarding diabetes management and meal planning**

1. The platform made me feel more confident in managing my diabetes

| 1 – Very strongly disagree | 2 | 3 | 4 | 5 – Neither agree nor disagree | 6 | 7 | 8 | 9 | 10 – Very strongly agree |
| --- | --- | --- | --- | --- | --- | --- | --- | --- | --- |
|  |  |  |  |  |  |  |  |  |  |

1. After using the platform, I felt more confident in making healthy food choices

| 1 – Very strongly disagree | 2 | 3 | 4 | 5 – Neither agree nor disagree | 6 | 7 | 8 | 9 | 10 – Very strongly agree |
| --- | --- | --- | --- | --- | --- | --- | --- | --- | --- |
|  |  |  |  |  |  |  |  |  |  |

1. After using the platform, I feel more confident planning meals

| 1 – Very unconfident | 2 | 3 | 4 | 5 – Neither confident nor unconfident | 6 | 7 | 8 | 9 | 10 – Very confident |
| --- | --- | --- | --- | --- | --- | --- | --- | --- | --- |
|  |  |  |  |  |  |  |  |  |  |

**Healthy eating**

1. Do you eat at least 2 portions (200g) of fruit everyday?

□ Always

□ Often

□ Sometimes

□ Never

1. Do you eat at least 2 portions (200g) of vegetables everyday?

□ Always

□ Often

□ Sometimes

□ Never

1. Your diet:

□ Is different everyday

□ Is different only sometimes during a week

□ Is different only during weekend days

□ Is very monotonous

1. Do you drink at least 1 – 1.5 litre mineral water every day?

□ Always

□ Often

□ Sometimes

□ Never

1. Your snacks are based mainly on:

□ Fruit/fruit juice and milk shakes/yoghurt

□ Biscuits/crackers/bread/stick bread

□ Fried patatos/popcorn/krapfen/peanuts/soft drinks

□ Sweets/chocolate/ice cream/cakes

1. Do you eat breakfast?

□ Always

□ Often

□ Sometimes

□ Never

1. How many times do you eat sweets and cakes in 1 week?

□ 1-2

□ 3-4

□ 1 time a day

□ more than 1 time daily

□ 1 time in 10-15 days

□ never

1. How many times do you eat fish in 1 week?

□ 1-2

□ 3-4

□ 1 time a day

□ more than 1 time daily

□ 1 time in 10-15 days

□ never

**System Usability Scale**

1. How often did you use the platform in the eight week period?

□ never

□ approximately once a month

□ approximately once a week

□ couple of times a week

□ every day

1. How many minutes did you use the platform approximately per session?

_____________ hours _____________ minutes


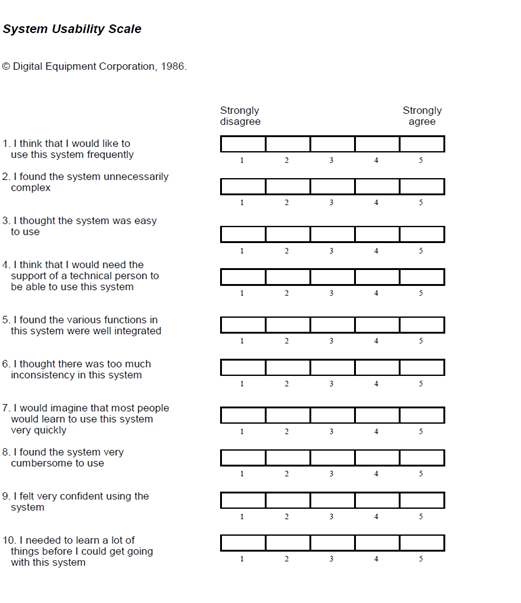


**Satisfaction**

1. The platform supported me in diabetes management

| 1 – Very Strongly disagree | 2 | 3 | 4 | 5 – Neither agree nor disagree | 6 | 7 | 8 | 9 | 10 – Very strongly agree |
| --- | --- | --- | --- | --- | --- | --- | --- | --- | --- |
|  |  |  |  |  |  |  |  |  |  |

Please explain your rating _________________________

1. The platform supported me in making healthy food choices?

| 1 – Very Strongly disagree | 2 | 3 | 4 | 5 – Neither agree nor disagree | 6 | 7 | 8 | 9 | 10 – Very strongly agree |
| --- | --- | --- | --- | --- | --- | --- | --- | --- | --- |
|  |  |  |  |  |  |  |  |  |  |

Please explain your rating _________________________

1. The platform supported me in planning my meals more efficiently and within budget?

| 1 – Very Strongly disagree | 2 | 3 | 4 | 5 – Neither agree nor disagree | 6 | 7 | 8 | 9 | 10 – Very strongly agree |
| --- | --- | --- | --- | --- | --- | --- | --- | --- | --- |
|  |  |  |  |  |  |  |  |  |  |

Please explain your rating _________________________

1. The platform supported me in my food shopping experience?

| 1 – Very Strongly disagree | 2 | 3 | 4 | 5 – Neither agree nor disagree | 6 | 7 | 8 | 9 | 10 – Very strongly agree |
| --- | --- | --- | --- | --- | --- | --- | --- | --- | --- |
|  |  |  |  |  |  |  |  |  |  |

Please explain your rating _________________________

5. Did the platform teach you anything else, apart from what is mentioned above?

□ Yes, please explain your answer _________________________

□ No, please explain your answer _________________________

6. Are there any features of this platform that you particularly liked?

□ Yes, please explain your answer _________________________

□ No, please explain your answer _________________________

7. Are there any features of this platform that you particularly disliked?

□ Yes, please explain your answer _________________________

□ No, please explain your answer _________________________

8. Did you encounter any technical problems?

□ Yes, please explain your answer _________________________

□ No, please explain your answer _________________________

9. The platform is a valuable addition to the Diabetes UK learning environment?

| 1 – Very Strongly disagree | 2 | 3 | 4 | 5 – Neither agree nor disagree | 6 | 7 | 8 | 9 | 10 – Very strongly agree |
| --- | --- | --- | --- | --- | --- | --- | --- | --- | --- |
|  |  |  |  |  |  |  |  |  |  |

Please explain your rating _________________________

10. The platform is easy to use / user-friendly

| 1 – Very Strongly disagree | 2 | 3 | 4 | 5 – Neither agree nor disagree | 6 | 7 | 8 | 9 | 10 – Very strongly agree |
| --- | --- | --- | --- | --- | --- | --- | --- | --- | --- |
|  |  |  |  |  |  |  |  |  |  |

Please explain your rating _________________________

11. Would you recommend the platform to other people who have Diabetes or are taking care of people with Diabetes?

□ Yes, please explain your answer _________________________

□ No, please explain your answer _________________________

12. How motivated were you to use the platform?

| 1 – Not very motivated | 2 | 3 | 4 | 5 – Neither unmotivated nor motivated | 6 | 7 | 8 | 9 | 10 – Very motivated |
| --- | --- | --- | --- | --- | --- | --- | --- | --- | --- |
|  |  |  |  |  |  |  |  |  |  |

Please explain your rating _________________________

13. Based on your experience, how would you rate the platform overall? (1=not good at all, 10 = very good)

| 1 | 2 | 3 | 4 | 5 | 6 | 7 | 8 | 9 | 10 |
| --- | --- | --- | --- | --- | --- | --- | --- | --- | --- |
|  |  |  |  |  |  |  |  |  |  |

14. Would you like to continue using the platform?

□ Yes, please explain your answer _________________________

□ No, please explain your answer _________________________

15. Do you think COVID-19 restrictions have been affecting optimal usage of the platform?

□ Yes, please explain your answer _________________________

□ No, please explain your answer _________________________

16. What would you improve to the platform?

………………………………………………………………………………………………………………………………………………………………………………………………………………………………………………………………………………………………………………………………………………………………………………………………............................................................................................................................................................................................................

**Thank you for filling in the second survey.**

If you agreed to participate into the prize draw you will find out through email if you won the first prize for participating in the second survey.

If you require further information please contact project lead Dr. Kim Bul on [kim.bul@coventry.ac.uk](mailto:kim.bul@coventry.ac.uk).

**21. Attachment_7: Invitation email to participate in an online case study interview (or phone interview alternatively)**

<Insert date>

Dear <Insert participant name>,

**Re: Invitation to participate in an online or telephone interview**

This invitation email has been sent to you after you agreed at the end of the first survey to be contacted by Coventry University for an online (or phone) case study interview for which you are eligible. You will be asked to share your experiences and perspectives, especially concerning the issues and challenges you encountered whilst using the platform. The interview will take up to 1.5 hours and will take place online.

Please find a Participant Information (PI) enclosed, which provides more details about the interview. Since you are eligible to participate, I would be grateful if you could read the PI and discuss this with your family and friends if you wish. If you agree to participate in this research activity, you can sign the enclosed Informed Consent Form (ICF) and send it back to me by email. If you prefer a phone interview, could you please indicate this in the email including your phone contact details? Once I received the signed ICF, I will confirm a date and time to suit you to perform the case study interview.

You are under no obligation to participate. There will be no consequences of not taking part. If you have any further questions, please do not hesitate to contact the project lead on [kim.bul@coventry.ac.uk](mailto:kim.bul@coventry.ac.uk)

I would like to thank you for your time and consideration.

Yours Sincerely,

Dr. Kim Bul

Nikki Holliday

Rachael Molitor

Coventry University

**22. Attachment_8: Participant Information (case study interview)**

**Project title**: **Platform evaluation**

**Project lead: Dr. Kim Bul**

**Local project team: Nikki Holliday, Rachael Molitor**

Dear Participant,

You are being invited to take part in research on the impact of the platform in supporting people with Diabetes or people who take care of people with this condition in their meal planning. Dr. Kim Bul, Research Fellow at Coventry University, is leading this research. Before you decide to take part, it is important you understand why the research is being conducted and what it will involve. Please take time to read the following information carefully.

**What is the purpose of the study?**

The aim of this study is to assess the impact of the platform, an online nutrition tool for people with Diabetes Type 1, Diabetes Type 2, who are at risk of developing Diabetes Type 2 or for people who are caring for a person with this condition, on several health and intervention outcomes and how often they are using the platform.

**Why have I been chosen to take part?**

The study is being conducted by Dr. Kim Bul at Coventry University. You have been selected to take part in an online (or phone) case study interview and agreed to be approached by us for this follow-up research activity.

**What are the benefits of taking part?**

This project provides you and other people who use the platform the opportunity to share your experiences. There will not be a direct benefit for you as an individual, but your answers will contribute to further development and improvement of the platform to support people with Diabetes Type 1, Diabetes Type 2, those who are at risk or taking care of someone with this condition.

**Are there any risk with taking part?**

This study has been reviewed and approved through Coventry University’s formal research ethics procedure (Insert ref number and date). There are no significant risks associated with participation. We ask that you answer all of the questions to aid our research, however if there are any questions you feel uncomfortable to answer, please let us know and we will go to the next questions.

**Do I have to take part?**

Your participation in the interview is entirely voluntary, and you can opt out at any stage by indicating this to the researcher.

**What will happen if I decide to take part?**

You will be asked to participate in an online (or phone)interview sharing your experiences of using the platform. No individual participants will be identified when the results are presented. Every participant will receive a final summary report of the results.

**Will my taking part in the research project be kept confidential?**

Your interview answers will be treated confidentially and the information you provide will be kept anonymous in any research outputs/publications. Your data will be processed in accordance with the General Data Protection Regulation 2016 (GDPR) and the Data Protection Act 2018. Video and audio recordings in Microsoft Teams will automatically be stored in Microsoft Stream and retained in password protected files which only Coventry University lead researchers have access to. Audio will be deleted from the recording device as soon as the recording is transferred to the allocated OneDrive folder. Video and audio recording will be deleted from the computer once transcribed. Personally identifiable data will be deleted 6 months after project start and anonymized data will be deleted three years after project start.

**What will happen if I don't want to carry on with the project?**

You are free to withdraw your case study interview responses from the project data set at any time until the data are fully anonymised in our records on [ENTER DATE]. You should note that your data may be used in the production of formal research outputs (e.g. journal articles, conference papers, theses and reports) prior to this date and so you are advised to contact the university at the earliest opportunity should you wish to withdraw from the study. To withdraw, please contact the lead researcher (contact details are provided below). Please also contact the Faculty Research Support Office (email hls.rso@coventry.ac.uk) telephone +44(0)2477658461) so that your request can be dealt with promptly in the event of the lead researcher’s absence. You do not need to give a reason. A decision to withdraw, or not to take part, will not affect you in any way.

**Data protection rights**

Coventry University is a Data Controller for the information you provide. You have the right to access information held about you. Your right of access can be exercised in accordance with the General Data Protection Regulation and the Data Protection Act 2018. You also have other rights including rights of correction, erasure, objection, and data portability. For more details, including the right to lodge a complaint with the Information Commissioner’s Office, please visit [www.ico.org.uk](http://www.ico.org.uk) Questions, comments and requests about your personal data can also be sent to the University Data Protection Officer - [enquiry.ipu@coventry.ac.uk](mailto:enquiry.ipu@coventry.ac.uk)

**Will I be reimbursed for taking part?**

You will receive a gift voucher of £20 as a thank you for giving up your time.

**Who is organising and funding the study?**

The West Midlands Academic Health Science Network (hosted by Birmingham NHS Foundation Trust) is funding the current research project.

**Further information and making a complaint**

For further information, or if you have any queries, please contact the lead researcher Dr. Kim Bul, [kim.bul@coventry.ac.uk](mailto:kim.bul@coventry.ac.uk). If you have any concerns that cannot be resolved through the lead researcher, please contact my linemanager Professor John Allen ([ad5325@coventry.ac.uk](mailto:ad5325@coventry.ac.uk)). Thank you for taking the time to participate in the case study interview. Your help is very much appreciated.

**23. Attachment_9: Informed Consent Form (case study interview)**

You are invited to take part in this research study for the purpose of collecting data on the efficacy and impact of the platform to support people with Diabetes or those whom take care of them.

Having read the Participant Information, please do not hesitate to ask questions if anything is unclear or if you would like more information about any aspect of this research (kim.bul@coventry.ac.uk). It is important that you feel able to take the necessary time to decide whether or not you wish to take part.

If you are happy to participate, please confirm your consent by clicking YES against each of the below statements and then signing and dating the form as participant. You will only be able to continue to the survey by selecting and agreeing to each of the statements below:

| **No.** | **Statement** | **NO** | **YES** |
| --- | --- | --- | --- |
| 1 | I confirm that I have read and understood the Participation Information for the study. I had the opportunity to consider the information, ask questions and have had these answered to my satisfaction. |  |  |
| 2 | I understand that my participation is voluntary and that I am free to withdraw before my information is anonymised on (DATE) without giving any reason, without my (medical) care or rights being affected. |  |  |
| 3 | I understand that all information I provide will be held securely and treated confidentially. |  |  |
| 4 | Should I choose to withdraw consent, I agree that information obtained from me in this research project up to that point may still be used (if not anonymized) |  |  |
| 5 | I agree to audio and video recording of the case study interview or audio recording of the phone interview alternatively, and the use of anonymized quotes in research reports and publications. |  |  |
| 6 | I am happy for the information I provide to be used (anonymously) in academic papers and other formal research outputs. |  |  |
| 7 | I agree to take part in this project. |  |  |

Please sign to say you agree to the above and wish to continue.

*When completed: 1 electronic copy for the participant and keep the original in the project OneDrive folder.*

**24. Attachment_10: Case study interview outline**

Thank you for agreeing to be interviewed. The interview will take about 1.5 hour with a small break in-between. The interview will focus on your experience with using the platform. The information you provide in this interview will be used to inform the evaluation of the platform, and may contribute to changes made to the platform. All the responses you provide will be anonymous in reports.

## To start…

1. What made you think it would be useful to use/take part?
2. How often did you use the platform?
3. How often did you use the cooking recipes?
4. How often did you use the shopping list activity?

## Instructions to use

1. Which device/devices did you download the platform on?
2. How did you find the instructions given on how to use the platform?
3. How easy were they to understand/read?
4. How helpful did you find these instructions?
5. What could have made them more helpful?

## Using the app

1. How easy did you find the platform to use?
   1. What made it easy?
   2. What was good about it?
   3. Did anything make it difficult to use?
   4. What was bad about it?
2. Did you try it on any other devices?
   1. Was one easier than the other? Why?
3. How easy did you find it to navigate around the platform?
4. What did you think about the ability to personalise what was relevant to you?
   1. Could anything be improved?
5. How did you find using the shopping list?
   1. Was it easy to navigate to/to find?
   2. Are there any changes/improvements that you would like to see?
6. How did you find using the cooking recipes?
   1. Was it easy to navigate to/to find?
   2. Are there any changes/improvements that you would like to see?

## Content

1. What did you think about the information that was included in the platform?
   1. Should there be more/less? Why?
   2. What did you think of the external links?
2. What did you think about the look/feel of the platform?
   1. Was it attractive?
   2. Were the pictures/images appropriate?
3. What did you think of the advice given to remedy the issues raised?

## Changes

1. Did you make any changes to your lifestyle or eating habits?
   1. If so, what changes and why?
      1. How do you feel now?
   2. If not, why not? (Costs, barriers, seen as unimportant?)
2. What did you do with the information provided in the platform?
3. Did you buy anything as a result?
4. Did you contact any other services?
5. Since using the app, do you feel any difference in terms of;
   1. Health
   2. Diabetes management
   3. Confidence
   4. Food shopping and budget
   5. Meal planning
   6. How do your family members/friends feel?

## COVID-19

1. Do you think COVID-19 restrictions have been affecting optimal usage of the platform? If so, please explain.

## Finishing

1. Do you have anything else you would like to say or add?

**25. Attachment_11: Invitation email to participate in an online semi-structured interview (or phone interview alternatively)**

<Insert date>

Dear <Insert participant name>,

**Re: Invitation to participate in an online semi-structured interview (or phone interview alternatively)**

This invitation email has been sent to you after you agreed at the end of the first survey to be contacted by Coventry University for a online (or phone) interview for which you are eligible. You will be asked to share your experiences and perspectives, including any issues and challenges you encountered whilst using the platform. The semi-structured interview will take approximately 1 hour and will take place online.

Please find a Participant Information enclosed, which provides more details about the interview. Since you are eligible to participate, I would be grateful if you could read the PI and discuss this with your family and friends if you wish. If you agree to participate in this research activity, you can sign the enclosed Informed Consent Form (ICF) and send it back to me by email. If you prefer a phone interview, could you please indicate this in the email including your phone contact details? Once I received the signed ICF, I will confirm an exact date and time to perform the case study interview.

You are under no obligation to participate. There will be no consequences of not taking part. If you have any further questions, please do not hesitate to contact the project lead on [kim.bul@coventry.ac.uk](mailto:kim.bul@coventry.ac.uk)

I would like to thank you for your time and consideration.

Yours Sincerely,

Dr. Kim Bul

Nikki Holliday

Racheal Molitor

Coventry University

**26. Attachment_12: Participant Information (semi-structured interview)**

**Project title**: **Platform evaluation**

**Project lead: Dr. Kim Bul**

**Local project team: Nikki Holliday, Rachael Molitor**

Dear Participant,

You are being invited to take part in research on the impact of using the platform in supporting people with Diabetes or people who take care of people with this condition in their meal planning. Dr. Kim Bul, Research Fellow at Coventry University, is leading this research. Before you decide to take part, it is important you understand why the research is being conducted and what it will involve. Please take time to read the following information carefully.

**What is the purpose of the study?**

The aim of this study is to assess the impact of the platform, an online nutrition tool for people with Diabetes Type 1, Diabetes Type 2, who are at risk of developing Diabetes Type 2 or for people who are caring for a person with this condition, on several health and intervention outcomes and how often they are using the platform.

**Why have I been chosen to take part?**

The study is being conducted by Dr. Kim Bul at Coventry University. You have been selected to take part in an online (or phone) interview and agreed to be approached by us for this follow-up research activity.

**What are the benefits of taking part?**

This project provides you and other people who have used the platform the opportunity to share your experiences with the platform. There will not be a direct benefit for you as an individual, but your answers will contribute to further development and improvement of the platform to support people with Diabetes Type 1, Diabetes Type 2, those who are at risk or taking care of someone with this condition.

**Are there any risk with taking part?**

This study has been reviewed and approved through Coventry University’s formal research ethics procedure (Insert ref number and date). There are no significant risks associated with participation. We ask that you answer all of the questions to aid our research, however if there are any questions you feel uncomfortable to answer, please let us know and we will go to the next questions.

**Do I have to take part?**

Your participation in the interview is entirely voluntary, and you can opt out at any stage by indicating this to the researcher.

**What will happen if I decide to take part?**

You will be asked to participate in an online (or phone) interview sharing your experiences and perspectives of using the platform. No individual participants will be identified when the results are presented. Every participant will receive a final summary report of the results.

**Will my taking part in the research project be kept confidential?**

Your interview answers will be treated confidentially and the information you provide will be kept anonymous in any research outputs/publications. Your data will be processed in accordance with the General Data Protection Regulation 2016 (GDPR) and the Data Protection Act 2018. Video and audio recordings in Microsoft Teams will automatically be stored in Microsoft Stream and retained in password protected files which only Coventry University lead researchers have access to. Audio will be deleted from the recording device as soon as the recording is transferred to the allocated OneDrive folder. Video and audio recording will be deleted from the computer once transcribed. Personally identifiable data will be deleted 6 months after project start and anonymized data will be deleted three years after project start.

**What will happen if I don't want to carry on with the project?**

You are free to withdraw your semi-structured interview responses from the project data set at any time until the data are fully anonymised in our records on [ENTER DATE]. You should note that your data may be used in the production of formal research outputs (e.g. journal articles, conference papers, theses and reports) prior to this date and so you are advised to contact the university at the earliest opportunity should you wish to withdraw from the study. To withdraw, please contact the lead researcher (contact details are provided below). Please also contact the Faculty Research Support Office (email hls.rso@coventry.ac.uk; telephone +44(0)2477658461) so that your request can be dealt with promptly in the event of the lead researcher’s absence. You do not need to give a reason. A decision to withdraw, or not to take part, will not affect you in any way.

**Data protection rights**

Coventry University is a Data Controller for the information you provide. You have the right to access information held about you. Your right of access can be exercised in accordance with the General Data Protection Regulation and the Data Protection Act 2018. You also have other rights including rights of correction, erasure, objection, and data portability. For more details, including the right to lodge a complaint with the Information Commissioner’s Office, please visit [www.ico.org.uk](http://www.ico.org.uk) Questions, comments and requests about your personal data can also be sent to the University Data Protection Officer - [enquiry.ipu@coventry.ac.uk](mailto:enquiry.ipu@coventry.ac.uk)

**Will I be reimbursed for taking part?**

You will receive a gift voucher of £10 to thank you for giving up your time.

**Who is organising and funding the study?**

The West Midlands Academic Health Science Network (hosted by Birmingham NHS Foundation Trust) is funding the current research project.

**Further information and making a complaint**

For further information, or if you have any queries, please contact the lead researcher Dr. Kim Bul, [kim.bul@coventry.ac.uk](mailto:kim.bul@coventry.ac.uk). If you have any concerns that cannot be resolved through the lead researcher, please contact my linemanager Professor John Allen ([ad5325@coventry.ac.uk](mailto:ad5325@coventry.ac.uk)). Thank you for taking the time to participate in the interview. Your help is very much appreciated.

**27. Attachment_13: Informed Consent Form (semi-structured interview)**

You are invited to take part in this research study for the purpose of collecting data on the efficacy and impact of the platform to support people with Diabetes or those whom take care of them.

Having read the Participant Information, please do not hesitate to ask questions if anything is unclear or if you would like more information about any aspect of this research (kim.bul@coventry.ac.uk). It is important that you feel able to take the necessary time to decide whether or not you wish to take part.

If you are happy to participate, please confirm your consent by clicking YES against each of the below statements and then signing and dating the form as participant. You will only be able to continue to the survey by selecting and agreeing to each of the statements below:

| **No.** | **Statement** | **NO** | **YES** |
| --- | --- | --- | --- |
| 1 | I confirm that I have read and understood the Participation Information for the study. I had the opportunity to consider the information, ask questions and have had these answered to my satisfaction. |  |  |
| 2 | I understand that my participation is voluntary and that I am free to withdraw at any time without giving any reason, without my (medical) care or rights being affected. |  |  |
| 3 | I understand that all information I provide will be held securely and treated confidentially. |  |  |
| 4 | Should I choose to withdraw consent, I agree that information obtained from me in this research project up to that point may still be used (if not anonymized). |  |  |
| 5 | I agree to audio and video recording of the semi-structured interview or audio recording of the phone interview alternatively, and the use of anonymized quotes in research reports and publications. |  |  |
| 6 | I am happy for the information I provide to be used (anonymously) in academic papers and other formal research outputs. |  |  |
| 7 | I agree to take part in this project. |  |  |

Please sign to say you agree to the above and wish to continue.

*When completed: 1 electronic copy for the participant and keep the original in the project OneDrive folder.*

**28. Attachment_14: Semi-structured interview for service users, platform staff and dieticians Diabetes UK**

Thank you for agreeing to be interviewed. The interview will take about 1 hour. The interview will focus on your experience with the platform. The information you provide in this interview will be used to inform the evaluation of the platform, and may contribute to changes made to the platform. All the responses you provide will be anonymous in reports.

## To start…

1. What made you think it would be useful to take part?
2. In what way are/were you involved with the platform?

Service users:

Tell me how you used the platform...

o What did you like about the platform?

o What did you not like about the platform?

o What was easy about using the platform?

o What was difficult about using the platform?

o Which part of the platform did you enjoy the most?

o What did you learn from the platform?

o Did the platform help you manage your diabetes?

o Why/why not/tell me more about how you used it to manage your diabetes

o What would you change about the platform?

o What would you keep the same?

o Do you have suggestions to improve the platform?

[Additional questions will be asked based on survey responses]

Diabetes UK staff/dietician:

o Did you enjoy the platform?

o Did you find it easy/difficult to use the platform?

o Which part of the platform did you enjoy the most?

o What did you learn from the platform?

o Do you have suggestions to improve the platform?

Platform developer staff:

o Did you enjoy being involved in setting-up the platform for Diabetes community?

o Did you find it easy/difficult to set-up the platform for Diabetes community?

o Which part of the platform did you enjoy the most to set-up for Diabetes community?

o What did you learn from the platform while setting-up for Diabetes community?

o Do you have suggestions to improve the platform for Diabetes community?

## Finishing

Do you have anything else you would like to say or add?

**29. Attachment_15: Transparency statement Diabetes UK**

*Diabetes UK transparency statement for web page*[*www.diabetes.org.uk/food-research*](https://eur01.safelinks.protection.outlook.com/?url=http%3A%2F%2Fwww.diabetes.org.uk%2Ffood-research&data=02%7C01%7Cac2658%40coventry.ac.uk%7C7e74e380062a4d86345708d83de6c2e9%7C4b18ab9a37654abeac7c0e0d398afd4f%7C0%7C0%7C637327408606108566&sdata=qIxy%2Bpcmb2brkglNECvtSwJ34rN7CpzKmj2tajRDXX0%3D&reserved=0)

Diabetes UK is partnering with Coventry University, platform developer and West Midlands Academic Health Science Network to research how people find, plan, and shop for food and how a recipe, meal planning and shopping tool might support people managing diabetes.

- Coventry University is carrying out the research.
- West Midlands Academic Health Science Network is providing the funding.
- The platform developer is creating the meal planning & shopping tool.

By clicking on this link, you are leaving to go to the platform website and entering a research environment. Please check the platform privacy policy when you click through for information on how your data will be stored.

Thank you for supporting this research.
