# Supplementary material for: Usability and Preliminary Efficacy of an Artificial Intelligence–Driven Platform Supporting Dietary Management in Diabetes: Mixed Methods Study
Source: JMIR Hum Factors. 2023 Aug 9;10:e43959. doi: 10.2196/43959 (PMC10448291; doi:10.2196/43959)
Supplement: Multimedia Appendix 3 [file humanfactors_v10i1e43959_app3.docx]

To start…

o What made you think it would be useful to take part?

o In what way are/were you involved with the platform?

Usability:

o Did you enjoy using the platform?

- Which part (or feature) of the platform did you enjoy the most? Examples of features are: shopping list, meal planner, recipes.
- Which part (or feature) of the platform did you dislike the most?

o Did you find it easy/difficult to use the platform?

- If easy, what made it easy?
- If difficult, what made it difficult? How would you change this?

Reach:

Do you think that the platform attracts and is user-friendly for people with Diabetes with different backgrounds (e.g., age, ethnicity, experience with computers and smartphones)?

Lifestyle changes:

o Did the platform support you in planning meals more efficiently?

o Did the platform support you in making healthy food choices?

o Did the platform support you in your Diabetes management (e.g., weight, waist size, blood glucose level, blood pressure level, cholesterol level)?

o Did the platform support you in your food shopping experience?

Value:

o What did you learn from the platform?

o What would you change about the platform?

o What would you keep the same?

o Do you have suggestions to improve the platform?

o Would you recommend the use of the platform to other people with Diabetes?

o Would the platform be a valuable addition to the Diabetes UK Learning Zone?

Uniqueness:

Are you aware of any similar applications for people with Diabetes?

COVID-19:

How do you think COVID-19 affected the usage of the platform among people with Diabetes?

Finishing

Do you have anything else you would like to say or add?
