# Supplementary material for: Usability and Preliminary Efficacy of an Artificial Intelligence–Driven Platform Supporting Dietary Management in Diabetes: Mixed Methods Study
Source: JMIR Hum Factors. 2023 Aug 9;10:e43959. doi: 10.2196/43959 (PMC10448291; doi:10.2196/43959)
Supplement: Multimedia Appendix 4 [file humanfactors_v10i1e43959_app4.docx]

|  | Diabetes experts | Platform users |
| --- | --- | --- |
| Recipes |  | - Include meat substitutes; - Include bread recipes; - Include more vegetarian recipes; - Improve readability of cooking instructions for recipes; - Present cooking time for all recipes (it is currently not always included); - Use British (instead of American) ingredients, or amend this per country. |
| Portion size | - Add an indication of portion size per person; - Add for how many persons each recipe is meant for; - Add more nutritional information on calories and carbohydrates; - Remove American cups (or define it clearly) across recipes as any confusion can lead to a different nutrition analysis and can have huge implications in terms of carbohydrates intake and blood glucose levels; - Offer recipes for single-person households; - Add weight for one serving (as a default setting) as people with diabetes often weigh their food; | - Define cups used across recipes; - Better explain how different serving sizes can be presented for each recipes; - Display nutritional value per 100g instead of per serving; - Add information on amount of carbohydrates per product and per serving; - Select 2 servings as the default setting as this better suits single person households; - Describe some ingredients more clearly (e.g., seltzer water). |
| Education and management | - Include education on what is healthy food; - Manage recipe content across communities; - Get recipes evaluated by an expert before they get published; - Offer instructions on what type of recipes are appropriate (for diabetes); - Add cooking instructions on meal preparation and freezer storage; - Add a counting tool for carbohydrate intake; - Mention that people always need to check-in with their healthcare professional before changing their diet; - Use the UK governmental traffic light labelling (will be updated soon). | - Add more educational information on diabetes and what to eat; - Review and manage recipe content; - Use example articles, social media or blogs to provide additional advice and support on health conditions; - Integrate platform with the NHS Dose Adjustment for Normal Eating (DAFNE) course; - Present health score for the entire meal plan (instead of individual meals); - Give specific examples on what foods to eat/avoid for people with diabetes; - Combine the platform with other educational resources (e.g., NHS, Diabetes UK) to create more awareness about what to eat (including nutritional value) and avoid; - Monitor diabetes related health outcomes through the platform (e.g., blood sugar levels); - Create some basic understanding on diabetes and food before using the   platform;   - Add a carbohydrate counting tool; - Use traffic light system for health score; - Add individual goal-setting. |
| Budget | - Add costs / family budget / price guide to each recipe; - Give suggestions on how to adapt recipes with healthy alternatives and affordable ingredients; - Add filter option on budget for recipes; - Include budget supermarkets into the online shopping list feature. | - Include the required budget across recipes; - Add additional shops for online shopping. |
| Peer support | - Add a chat function so people can share tips and experiences; - Add in demonstrations / videos from different communities on how they manage their diabetes; - Add functionality to transfer shopping list to a person who supports people with learning difficulties. |  |
| Tailored content | - Personalise and individualize to allow “pushing” of content into the direction of the user preferences; - People need to feel that the platform suits their needs; - Offer clear instructions on how to use the platform and what the benefits are; - Do not make the platform too reliant on the individual to act and provide enough guidance and stimulation; - Do not offer too many options /choices as people will not explore further and only stick with one or two options; - Add notifications for people about topics they find interesting to enhance user engagement; - Personalise the platform by acting on insights gained through user behaviour; - Add a personalised dashboard allowing the user to pin recipes and meal plans; - Add an example of a meal plan that represents the total allowed carbohydrates per day as a starting point; - Add personalised goal setting and budget to stimulate meal planner usage; - Present glycaemic index (GI) and load (GL) separately for diabetes Type 1 and Type 2 as GI seems more relevant for Type 2 and GL more relevant for Type 1; - Add recipe information (such as GL and GI) where relevant and not across all communities; - Improve filter functionality for recipes (e.g., distinguish vegan and vegetarian). | - Display an option to amend carbohydrates across recipes; - Embed more personalized content for people with Type 1 diabetes; - Allow sorting the shopping list by food groups such as vegetables, fruits etc.; - Present recipes according to type of meal (e.g., breakfast, lunch, dinner, snack, dessert) or ingredients; - Divide meal planner into breakfast, lunch and dinner categories; - Integrate with functionality to monitor physical activity. |
| Lay-out | - Make the content more readable, user-friendly and accessible for people with visual impairments and older people in particular; - Make filter and search bar functionalities more noticeable to enhance platform navigation; - Cluster recipes and give them different headings (for example by meal type); - Make the option to change background colour and font size more noticeable; - Give the homepage a calmer and less busy interface; - Meal planner should be visible in the banner on the homepage; - Try to avoid text over images as this is not user-friendly for people with diabetes who suffer from visual impairments. | - Embed a directly visible help or FAQs button on top of the bar at the homepage. |
